# Proximal Tubule mTORC1 Is a Central Player in the Pathophysiology of Diabetic Nephropathy and Its Correction by SGLT2 Inhibitors

**DOI:** 10.1016/j.celrep.2020.107954

**Published:** 2020-07-28

**Authors:** Aviram Kogot-Levin, Liad Hinden, Yael Riahi, Tal Israeli, Boaz Tirosh, Erol Cerasi, Ernesto Bernal Mizrachi, Joseph Tam, Ofri Mosenzon, Gil Leibowitz

**Affiliations:** 1 Diabetes Unit and Endocrine Service, Hadassah-Hebrew University Medical Center, Jerusalem, Israel; 2 Obesity and Metabolism Laboratory, Institute for Drug Research, School of Pharmacy, Faculty of Medicine, The Hebrew University of Jerusalem, Jerusalem, Israel; 3 Stress Signaling Laboratory, School of Pharmacy, The Hebrew University of Jerusalem, Jerusalem, Israel; 4 Department of Internal Medicine, Division of Endocrinology, Metabolism and Diabetes, Miller School of Medicine, University of Miami, Miami, FL, USA

**Keywords:** diabetes, complications, diabetic kidney disease, signal transduction, SGLT2 inhibitors, mTOR, fibrosis

## Abstract

Diabetic kidney disease (DKD) increases the risk for mortality and is the leading cause of end-stage renal disease. Treatment with sodium-glucose cotransporter 2 inhibitors (SGLT2i) attenuates the progression of DKD, especially in patients with advanced kidney disease. Herein, we show that in diabetes, mTORC1 activity is increased in renal proximal tubule cells (RPTCs) along with enhanced tubule-interstitial fibrosis; this is prevented by SGLT2i. Constitutive activation of mTORC1 in RPTCs induces renal fibrosis and failure and abolishes the renal-protective effects of SGLT2i in diabetes. On the contrary, partial inhibition of mTORC1 in RPTCs prevents fibrosis and the decline in renal function. Stimulation of mTORC1 in RPTCs turns on a pro-fibrotic program in the renal cortex, whereas its inhibition in diabetes reverses the alterations in gene expression. We suggest that RPTC mTORC1 is a critical node that mediates kidney dysfunction in diabetes and the protective effects of SGLT2i by regulating fibrogenesis.

## Highlights

• In diabetes, mTORC1 activity is increased in renal proximal tubule cells (RPTCs)
• Diabetes and SGLT2i regulate mTORC1 by modulating nutrient transport to RPTCs
• Inhibition of mTORC1 in RPTCs prevents fibrosis and the decline in renal function
• RPTC mTORC1 mediates renal fibrosis in diabetes and the beneficial effects of SGLT2i

## Graphical Abstract

**Figure:**
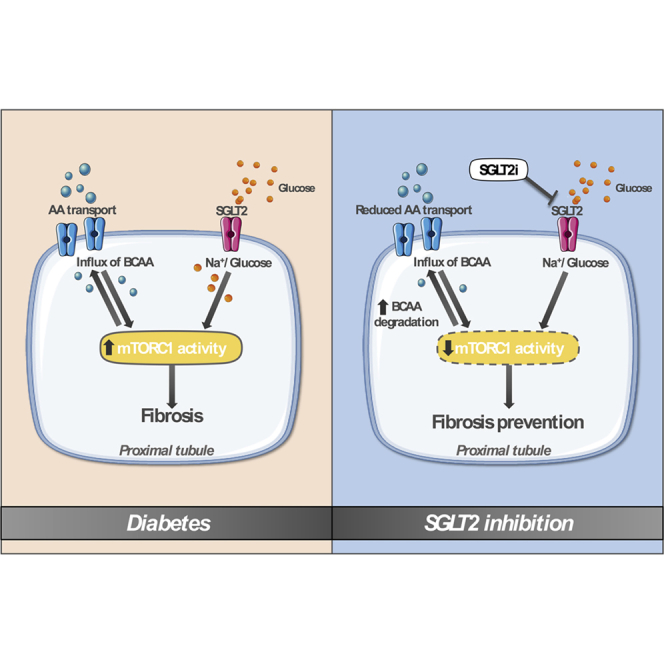

## Teaser

Kogot-Levin et al. show that treatment with sodium-glucose cotransporter 2 inhibitors (SGLT2i) attenuates the progression of diabetic kidney disease (DKD), which is the leading cause of end-stage renal disease. The nutrient sensor mTORC1 is a critical node that mediates kidney dysfunction in diabetes and the protective effects of SGLT2i by regulating fibrogenesis.

## Introduction

Chronic kidney disease (CKD) is one of the most common complications of diabetes, occurring in 20%–40% of patients (Afkarian et al., 2016; de Boer and Group, 2014; Ni et al., 2017). Diabetic kidney disease (DKD) is diagnosed by the persistent presence of elevated urinary albumin excretion and/or low glomerular filtration rate (Tuttle et al., 2014). DKD can progress to end-stage renal disease (ESRD) requiring dialysis or kidney transplantation; it is the leading cause of ESRD in the Western world (Lytvyn et al., 2020). In addition, among people with type 1 or 2 diabetes, the presence of CKD markedly increases cardiovascular risk and health care costs (Fox et al., 2012).

Hitherto, the accepted paradigm has been that DKD is initially a glomerular disease manifested by hyperfiltration and gradual loss of glomerular charge and permselectivity, leading to albuminuria followed by renal failure (Tonneijck et al., 2017). These alterations are associated with typical ultrastructural changes, including glomerular hypertrophy and glomerulosclerosis. However, in many diabetic subjects renal failure is not preceded by increasing albuminuria, the hallmark of glomerular disease; in fact, non-albuminuric renal failure is now becoming the predominant form of DKD (Piscitelli et al., 2017; Zeni et al., 2017).

Gilbert and Cooper highlighted the existence of non-glomerular mechanisms involving tubular damage, which may have an important role in the pathophysiology of DKD (Gilbert, 2017; Gilbert and Cooper, 1999). Moreover, structural changes in the tubular region, including tubular atrophy, interstitial fibrosis, and peritubular capillary rarefaction, correlate with declining kidney function in patients with DKD (Gilbert and Cooper, 1999; Zeni et al., 2017). Recently, sodium-glucose cotransporter 2 inhibitors (SGLT2i), which inhibit glucose uptake by the renal proximal tubule cells (RPTCs), revolutionized the treatment of DKD. Large-scale, randomized, controlled prospective clinical trials consistently showed that SGLT2i are highly effective in preventing the decline of kidney function in diabetic patients and the progression to ESRD, including and probably even more so in patients with advanced renal disease (Mosenzon et al., 2019; Perkovic et al., 2019; Wanner et al., 2016; Zelniker et al., 2019). The current dogma is that the beneficial effect of SGLT2i is primarily a nephron-hemodynamic effect, which is mediated via the tubuloglomerular feedback that leads to relative arteriolar vasoconstriction and consequently reduction of glomerular filtration pressure (Kidokoro et al., 2019; Thomson et al., 2012; Vallon et al., 2013); however, this hypothesis has not been verified in more recent human studies (van Bommel et al., 2020). Additional effects, including reduction of hypoxia, inflammation, and serum uric acid toxicity, may have a role in renal protection by SGLT2i (Heerspink et al., 2018, 2019; Lytvyn et al., 2015; van Raalte and Cherney, 2018). In short, the precise molecular mechanisms underlying the robust renal-protective effects of SGLT2i are still unclear.

RPTCs are anatomically localized close to the glomerulus and specialize in sodium-glucose linked transport to enable effective reabsorption of sodium for the maintenance of fluid homeostasis. Remarkably, 60% of the kidney’s energy consumption is devoted to sodium reabsorption, with RPTCs accounting for approximately two-thirds of it, primarily due to basal Na^+^/K^+^ ATPase activity (Singh et al., 2016). RPTCs rely on oxidative phosphorylation for ATP generation required for glucose-sodium transport. In diabetes, the sodium reabsorption workload on RPTCs is increased by hyperglycemia, most likely via increased SGLT2 expression in these cells (Wang et al., 2017; Rahmoune et al., 2005; Vestri et al., 2001), which in turn elevates the glomerular filtration of glucose followed by its reuptake in RPTCs (Gilbert, 2017). We hypothesize that this leads to sustained activation of nutrient-sensing pathways in RPTCs, which may promote the development and progression of DKD.

The rapamycin-sensitive complex of mTOR, mTORC1, integrates signals from nutrients, mainly glucose and branched-chain amino acids (BCAAs), to control protein synthesis, cell size, proliferation, and autophagy (Kim and Guan, 2019). Herein, we show that activation of mTORC1 is an early culprit in the course of DKD, which promotes fibrogenesis. Treatment with SGLT2i inhibited mTORC1 and prevented kidney dysfunction. We further show that genetic activation of mTORC1 *in vivo* mimicked the alterations of DKD and abrogated the protective effects of SGLT2i, whereas genetic inhibition of mTORC1 mirrored the effects of SGLT2i and prevented fibrogenesis and renal failure. Collectively, these findings suggest that RPTC mTORC1 plays a key role in the pathophysiology of DKD and in mediating the beneficial effects of SGLT2i.

## Results

### SGLT2i Prevents Diabetic Kidney Disease

The *Akita* mouse is a common model for studying DKD (Kitada et al., 2016). These mice develop insulin-deficient diabetes at young age because of β-cell stress, reminiscent of human type 1 diabetes (T1D). We treated 2-month-old *Akita* mice with dapagliflozin (a SGLT2i) (10 mg/kg/day) added to drinking water for 12 weeks (experimental design shown in Figure S1A) and tested the effects on kidney function and morphometry. *Akita* mice had marked hyperglycemia and gained less body weight compared with age-matched normoglycemic control mice (Figures S1B and S1C). Treatment of *Akita* mice with dapagliflozin normalized blood glucose without affecting body weight, along with increased urinary excretion of glucose and sodium compared with wild-type mice (Figures S1B–S1E). The urine glucose excretion was lower than that of diabetic *Akita* mice, because treatment with dapagliflozin normalized blood glucose, thereby reducing glucose concentration in the glomerular filtrate. Serum insulin levels were markedly decreased in the *Akita* mice and were not affected by treatment with dapagliflozin (Figure S1F). In addition, serum β-hydroxybutyrate and BCAA levels were not increased in diabetic animals treated with or without dapagliflozin (Figures S1G and S1H). These findings are consistent with the known mechanism of action of SGLT2i, which improves diabetes by inhibiting renal glucose-sodium absorption, and show that residual insulin secretion was sufficient to inhibit ketogenesis, including in the presence of SGLT2i.

Of note, *Sglt2* but not *Sglt1* gene expression was increased in diabetes, whereas treatment with dapagliflozin decreased both *Sglt2* and *Sglt1* expression (Figure S1I). The changes in diuresis and water intake mirrored glucosuria (Figures S1J and S1K). The kidney weight of *Akita* mice was higher than that of wild-type mice; kidney enlargement was not affected by treatment with dapagliflozin (Figure S1L). *Akita* mice developed DKD, evident by albuminuria, increased serum creatinine and blood urea nitrogen (BUN) levels, decreased creatinine clearance, and increased urinary excretion of KIM-1, a marker of tubular injury. Treatment with dapagliflozin decreased albuminuria and KIM-1 excretion and prevented the decline of creatinine clearance (Figures S1M–S1Q).

**Figure 1:**
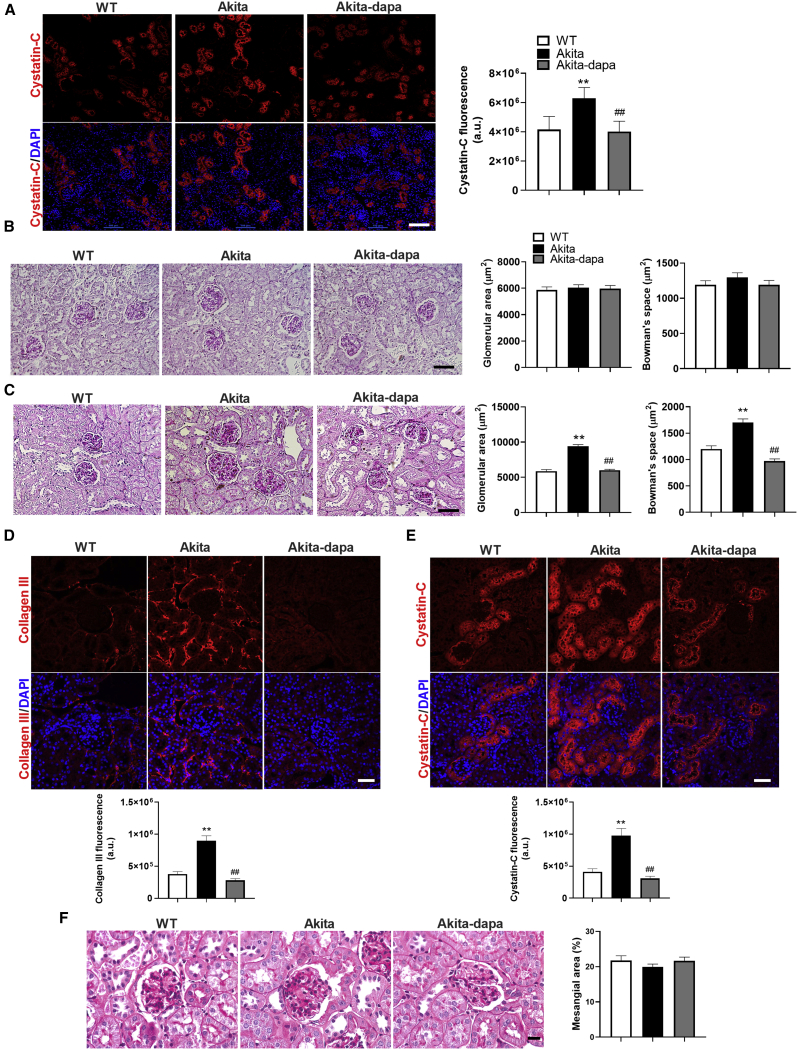
Effects of Short- and Long-Term Treatment with SGLT2i on Glomerular Size, Renal Injury, and Tubule-Interstitial Fibrosis Akita mice aged 7–8 weeks were treated with and without dapagliflozin (10 mg/kg/day) for 5 days or 3 months. (A) Immunofluorescence staining for the tubule injury marker cystatin-C at the age of 2 months. (B and C) Representative periodic acid-Schiff (PAS) staining and quantifications of glomerular and Bowman’s space cross-sectional areas at the ages of 2 months (B) and 5 months (C). (D and E) Immunofluorescence staining for the fibrosis marker collagen III (D) and the proximal tubule injury marker cystatin-C (E). Representative images and quantification of fluorescence intensity are shown. (F) Representative PAS staining of the glomeruli and quantification of PAS-positive percentage of mesangial area. Scale bar, 50 μm. Data represent the mean ± SEM of six to eight mice per group. ^∗∗^p < 0.01 relative to the wild-type control group; ^##^p < 0.01 relative to the untreated Akita mice group.

Immunostaining of kidney sections of 8-week-old diabetic *Akita* mice showed increased expression of the tubular injury marker cystatin-C compared with wild-type control mice (Figure 1A). Short-term (5 days) treatment with dapagliflozin reversed the increase in cystatin-C (Figure 1A). Staining for collagen III and collagen Ι showed no evidence for interstitial fibrosis at this stage (not shown). Moreover, there was no glomerular hypertrophy in the diabetic animals, and glomerular size was not affected by treatment with dapagliflozin (Figure 1B). These findings suggest that tubular injury precedes the development of full-blown DKD and can be rapidly reversed by treatment with SGLT2i. We then studied the long-term effects of treatment with SGLT2i on the development of DKD. After an additional 12 weeks, diabetic mice developed marked glomerular hypertrophy, evident by an increase of ∼50% in glomerular and Bowman’s space areas (Figures 1C–1E). In addition, there was marked peritubular fibrosis, along with increased expression of cystatin-C (Figures 1D and 1E); these alterations were prevented by the 3-month treatment with dapagliflozin (Figures 1D and 1E). There was no mesangial expansion in the glomeruli of diabetic animals, and treatment with dapagliflozin did not affect the mesangial area (Figure 1F). Consistently, moderate glomerular mesangial expansion, without podocyte effacement, has been reported in aged but not in young *Akita* mice (Chang et al., 2012).

Collectively, these results show that in *Akita* mice, diabetes induced progressive morphological changes in the kidney with subsequent development of full-blown DKD, including albuminuria and renal failure, along with glomerular hypertrophy and interstitial fibrosis. The tubular changes preceded the alterations in glomerular size and were prevented by treatment with dapagliflozin.

### mTORC1 Activity Is Increased in RPTCs in Diabetes and Inhibited by SGLT2i

**Figure 2:**
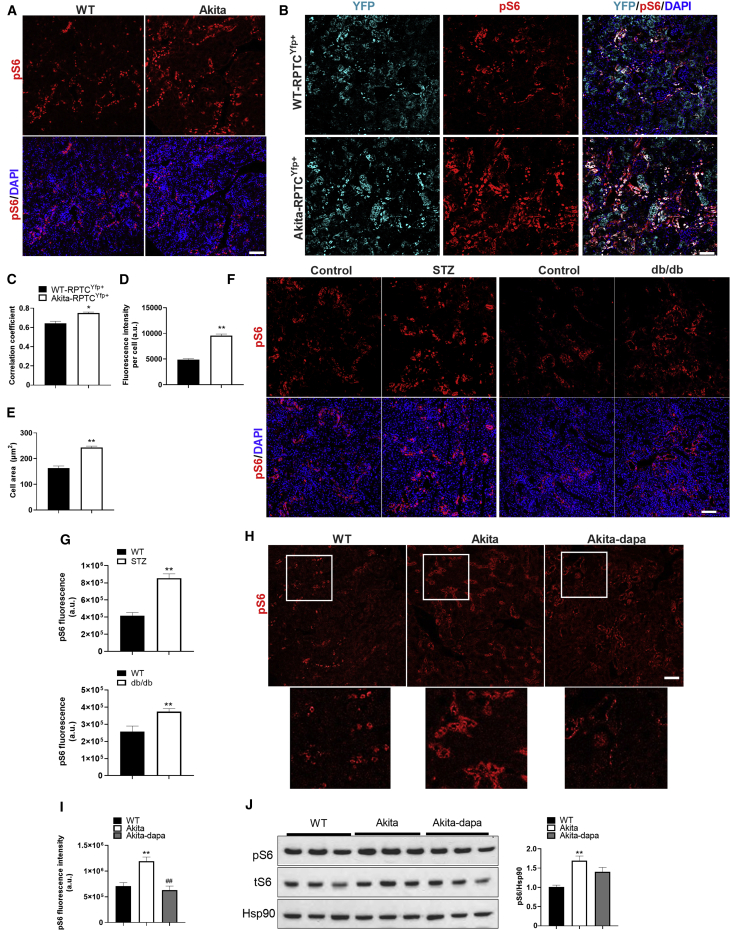
Effects of Diabetes and of Treatment with SGLT2i on RPTCs mTORC1 Activity (A) Immunofluorescence staining for pS6 on kidney sections of 2-month-old wild-type and *Akita* mice. (B) Immunofluorescence staining for YFP and pS6 in lineage-traced *Sglt2-Cre;Rosa26-YFP*^*+*^ reporter wild-type and *Akita* mice. (C) Correlation coefficient of YFP and pS6 co-expression. (D and E) pS6 fluorescence intensity (D) and (E) quantifications of RPTC area. (F and G) Immunofluorescence staining for pS6 on kidney sections of streptozotocin (STZ)-induced diabetic mice and in db/db mice (F). Quantification of pS6 fluorescence intensity is shown in (G). (H and I) Immunofluorescence staining for pS6 on kidney sections of wild-type and *Akita* mice treated with and without dapagliflozin for 12 weeks (H). Insets shown below are higher magnification of the area surrounded by a square; quantifications are shown in (I). (J) Western blotting for pS6 on whole-kidney extracts of wild-type and *Akita* mice treated with and without dapagliflozin. Scale bar, 50 μm. Data represent the mean ± SEM of six to eight mice per group. ^∗^p < 0.05 and ^∗∗^p < 0.01 relative to the wild-type control group; ^##^p < 0.01 relative to the untreated *Akita* mice group.

Next, we tested the hypothesis that mTORC1 activation in RPTCs plays a role in the pathophysiology of DKD. To this end, we studied the localization and activity of mTORC1 in kidney sections of wild-type and diabetic *Akita* mice. We assessed mTORC1 activity by immunostaining for phospho-S6 and found that in both control and diabetic animals, mTORC1 activity (pS6^+^ cells) is localized mainly in the tubular regions of the kidney, with relatively faint staining in the glomerulus (Figure 2A). There was strong co-localization of pS6 and the proximal tubule glucose transporter 2 (GLUT2; correlation coefficient ∼0.7) (Figure S2A), suggesting that mTORC1 is active in RPTCs. To verify this assumption, we analyzed pS6 expression in lineage-traced RPTCs (YFP^+^ cells). For this purpose, we generated *Sglt2-Cre*; *Rosa26-YFP* reporter mice on the background of wild-type and *Akita* mice (Figure S2B). RPTCs are contiguous with the Bowman’s capsule. Consistently, YFP^+^ cells were observed in the Bowman’s capsule and in adjacent tubules (Figure S2C). We did not find any YFP^+^ cells in the liver, small intestine, and pancreatic islets, indicating that lineage tracing was quite specific for RPTCs (Figures S2D–S2F). RPTCs express the cell adhesion molecule N-cadherin, whereas distal tubular cells express predominantly E-cadherin (Nouwen et al., 1993). We found no co-localization of YFP and E-cadherin, indicating that lineage tracing was restricted to RPTCs, without leakiness of labeling to the distal parts of the nephron (Figure S2G). In wild-type mice, there was strong co-localization of pS6 and YFP in RPTCs, with a correlation coefficient of ∼0.6; this was further increased in the diabetic animals (Figures 2B and 2C). pS6 fluorescence intensity was increased in diabetic *Akita* mice, accompanied by marked increase of cell size in RPTCs, resulting in tubular hypertrophy (Figures 2D and 2E). The pS6 fluorescence intensity was also increased in RPTCs of streptozotocin (STZ)-induced diabetic mice (T1D model), and in *db*/*db* mice (T2D model), further supporting activation of mTORC1 in diabetes (Figures 2F and 2G). RPTC proliferation (percentage of Ki67^+^/YFP^+^ cells) was decreased in *Akita* diabetes despite stimulation of mTORC1, whereas treatment with dapagliflozin prevented this decrease (Figure S2H). Lineage-traced RPTCs showed no increase in the expression of the mesenchymal protein α-smooth muscle actin (αSMA) or downregulation of the epithelial marker Zona occludens-1 (ZO-1), indicating that diabetes was not associated with epithelial-mesenchymal transition of RPTCs (Figures S3A and S3B).

Treatment of diabetic *Akita* mice with dapagliflozin decreased mTORC1 activity (pS6 fluorescence intensity) in RPTCs (Figures 2H and 2I). Western blotting of whole-kidney homogenates also showed that mTORC1 activity (both phospho- and total S6) was increased in *Akita* mice; this was partially decreased by dapagliflozin (Figure 2J). The limited decrease of mTORC1 activity by western blotting in dapagliflozin-treated animals is probably due to the use of whole-kidney homogenate that contains mainly cells other than RPTCs. Sustained activation of mTORC1 might suppress PI3 kinase/AKT activity through phosphorylation and increased degradation of insulin receptor substrate (IRS) proteins and/or inhibition of mTORC2, which is required for AKT phosphorylation at serine 473 (Ardestani et al., 2018). Indeed, we found that pAKT(S473) was decreased in RPTCs of diabetic *Akita* mice; this was prevented by treatment with dapagliflozin (Figure S3C).

**Figure 3:**
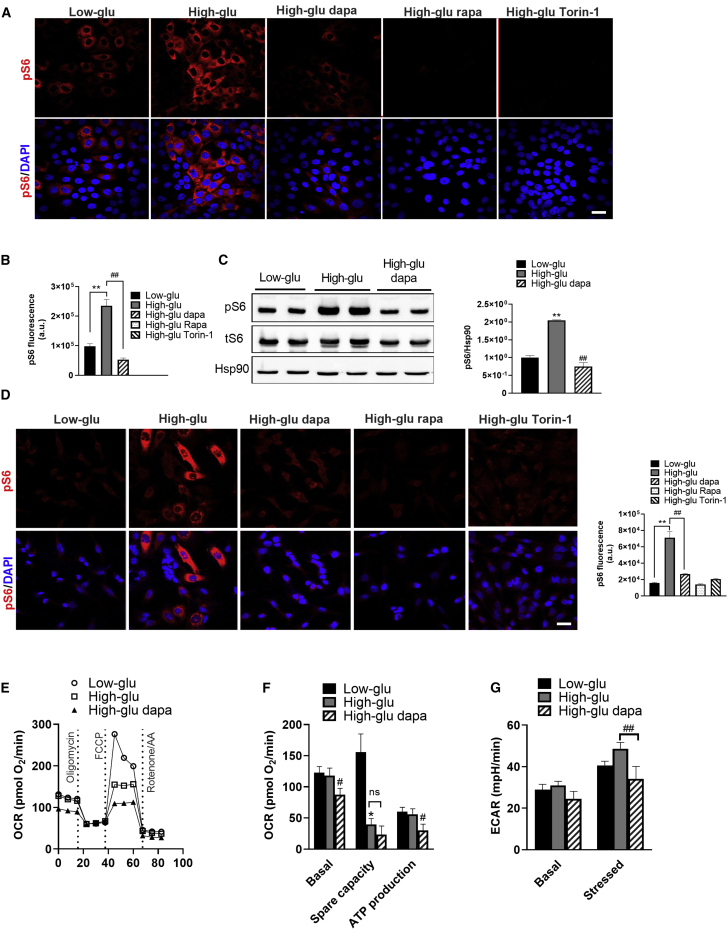
Effects of High Glucose and SGLT2i on mTORC1 Activity, Glycolysis, and Mitochondrial Respiration Cultured RPTCs (LLC-PK1 and HK2) were incubated at 5 mM glucose (low-glu) or 30 mM glucose (high-glu) with or without dapagliflozin (5 μM) for 48 h. The mTORC1 inhibitors rapamycin (rapa) and Torin-1 were used as controls. (A and B) Immunofluorescence staining for pS6 (A) and quantification of fluorescence intensity (B) in LLC-PK1 cells. (C) Western blotting for pS6 in LLC-PK1 cells. (D) Immunofluorescence staining for pS6 and quantification of fluorescence intensity in HK2 cells. (E–G) Oxygen consumption rate (OCR) (E and F) and (G) extracellular acidification rate (ECAR) in LLC-PK1 cells. Scale bar, 50 μm. Data represent the mean ± SEM of three independent experiments. ^∗^p < 0.05 and ^∗∗^p < 0.01 relative to cells incubated at low glucose; ^#^p < 0.05 and ^##^p < 0.01 relative to the high-glucose group.

Dapagliflozin may reduce mTORC1 activity either by systemic correction of the hyperglycemia or locally by blocking Na^+^/glucose transport into RPTCs. To assess the effect of blood glucose regulation, we treated diabetic *Akita* mice with insulin degludec or dapagliflozin for 5 days; insulin and dapagliflozin similarly reduced glycemia (Figure S4A). Dapagliflozin robustly decreased mTORC1 activity in RPTCs; on the contrary, insulin increased mTORC1 activity, along with decreased AKT phosphorylation at serine 473 (Figures S4B and S4C). We further performed *in vitro* studies in LLC-PK1 (pig) and HK2 (human) RPTC cell lines and showed that whereas incubation of RPTCs at high glucose (30 mM) increased mTORC1 activity, treatment with dapagliflozin or the mTORC1 inhibitors rapamycin or Torin1 prevented glucose stimulation of mTORC1 (Figures 3A–3D). We analyzed the effects of high glucose and of dapagliflozin on glycolysis and mitochondrial activity by Seahorse in LLC-PK1 cells. Mitochondrial maximal respiration capacity was reduced following 24 h exposure to 30 mM glucose, probably because of glucotoxicity; this was not rescued by dapagliflozin treatment (Figures 3E and 3F). Dapagliflozin decreased basal mitochondrial respiration and respiration-coupled ATP production (Figure 3F) and glycolysis (Figure 3G). Collectively, these findings indicate that SGLT2i reduce the workload on RPTCs by inhibiting both glucose oxidation and glycolysis.

In summary, these findings show that mTORC1 activity is high in RPTCs compared with other regions of the kidney and is further increased in diabetes. Treatment with SGLT2i decreases mTORC1 activity in RPTCs by reducing their glucose transport and metabolism.

### Constitutive Activation of mTORC1 in RPTCs Induces Kidney Dysfunction, Albuminuria, and Interstitial Fibrosis

**Figure 4:**
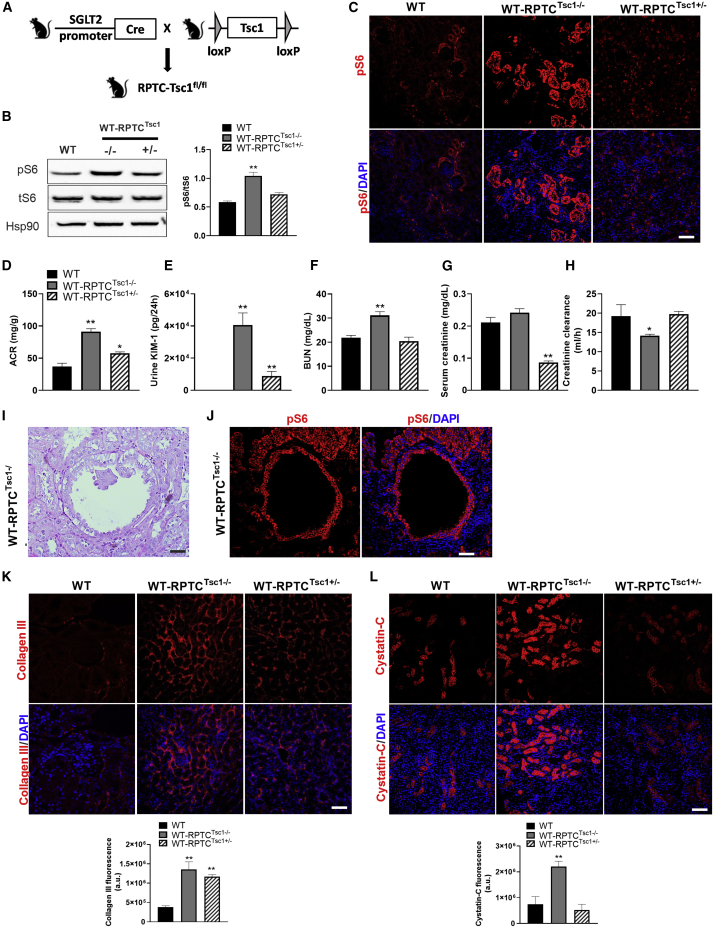
Effects of Constitutive Activation of mTORC1 in RPTCs on Renal Function, Albuminuria, and Interstitial Fibrosis (A) Generation of conditional *RPTC-Tsc1*-KO mice. (B and C) Western blotting (B) and immunostaining for pS6 (C). Quantification of pS6 expression is shown in (B). (D–H) Parameters of kidney injury and function: (D) urine ACR, (E) urine KIM-1 levels, (F) serum creatinine, (G) BUN, and (H) creatinine clearance. (I and J) Generation of renal cysts in *RPTC-Tsc1*-KO mice. A representative PAS staining is shown in (I) and immunofluorescence staining for pS6 in (J). (K and L) Immunofluorescence staining and quantification of collagen III (K) and cystatin-C (L). Scale bar, 50 μm. Data represent the mean ± SEM of four mice per group. ^∗^p < 0.05 and ^∗∗^p < 0.01 relative to the wild-type control group.

To distinguish causative links between mTORC1 and DKD from correlative variation, we studied the effect of increased mTORC1 activity in RPTCs by deleting *Tsc1*, an essential component of the tuberous sclerosis complex, which inhibits mTORC1 through phosphorylation of Rheb. We generated *Sglt2-Cre*;*Tsc1*^*fl/fl*^ and *Sglt2-Cre*;*Tsc1*^*fl/+*^ knockout (KO) mice (Figure 4A) and studied the effects on kidney morphometry and function. Knocking out *Tsc1* in RPTCs stimulated mTORC1, evident by increased pS6 expression (immunostaining and western blotting; Figures 4 and 4C). RPTC-*Tsc1* KO did not affect glycemia (Figure S5A). The body weight of *RPTC-Tsc1*-KO mice was lower than that of controls, resulting in increased kidney/body weight ratio (Figures S5B and S5C). Surprisingly, we found that *RPTC-Tsc1* KO enhanced glucosuria, diuresis, and consequently water intake in these non-diabetic mice (Figures S5D–S5F); we suggest that low body weight is due to life-long glucosuria, leading to energy waste. Inhibition of glucose transport into RPTCs is expected to increase glucose and sodium transport to the macula densa, with subsequent activation of the tubuloglomerular feedback. Consistently, we found that glomerular size was indeed reduced in *RPTC-Tsc1*-KO mice (Figures S5G and S5H). The urinary excretion of sodium, calcium, phosphate, and uric acid was not enhanced in *RPTCs Tsc1*-KO mice compared with wild-type mice (Figures S5I–S5L). Collectively, these findings suggest that constitutive activation of mTORC1 alters glucose transport in RPTCs.

Homozygous *Tsc1* KO in RPTCs increased albuminuria and urinary KIM-1 excretion and induced renal failure (Figures 4D–4H). In addition, some of the animals developed large renal cysts that were lined with pS6^+^ cells (Figures 4I and 4J). Immunohistochemistry further showed increased expression of cystatin-C and induction of interstitial fibrosis (collagen III) (Figures 4K and 4L). Notably, heterozygous KO of *Tsc1* in RPTCs was sufficient to induce peritubular fibrosis, indicating that mTORC1 activity is strongly associated with fibrosis.

Collectively, these findings suggest that activation of mTORC1 in RPTCs induces tubular dysfunction, peritubular fibrosis, albuminuria, and kidney dysfunction, mimicking the findings in diabetic *Akita* mice, despite the absence of hyperglycemia.

### Constitutive Activation of mTORC1 in RPTCs Abrogates the Renal-Protective Effect of SGLT2i

**Figure 5:**
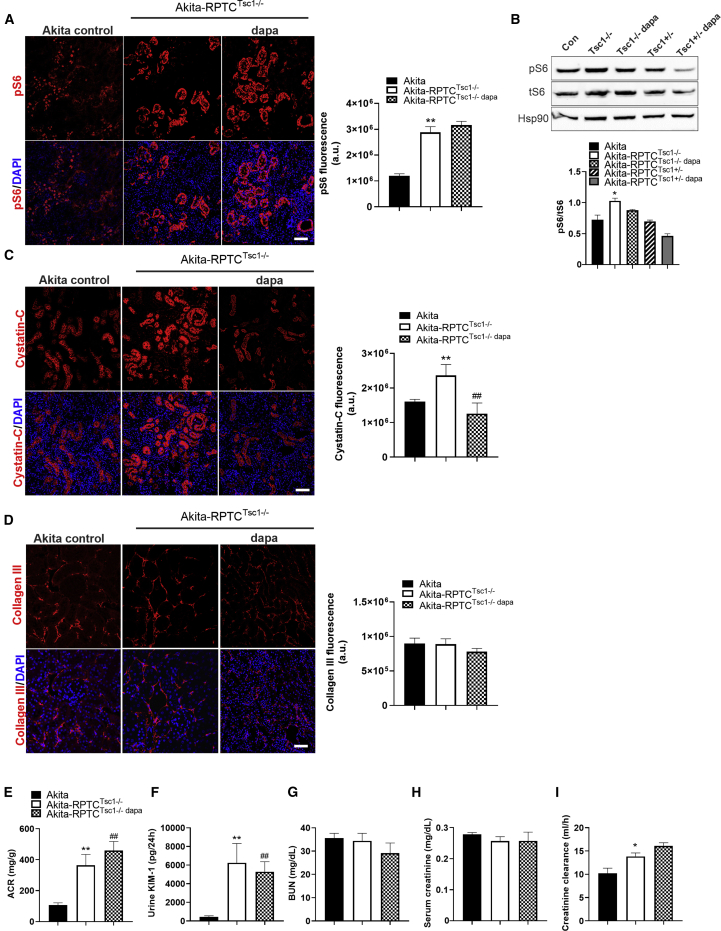
Activation of mTORC1 in *Akita* RPTCs Abrogates the Renal-Protective Effect of SGLT2i *RPTC-Tsc1*-KO *Akita* mice were treated with or without dapagliflozin (10 mg/kg/day in drinking water) for 12 weeks and compared with control *Akita* mice. (A and B) pS6 expression by immunofluorescence (A) and by western blotting (B). (C and D) Immunofluorescence for cystatin-C (C) and (D) for collagen III. (E–I) Parameters of kidney injury and function: urine ACR (E), urine KIM-1 levels (F), BUN (G), serum creatinine (H), and creatinine clearance (I). Scale bar, 50 μm. Data represent the mean ± SEM of three or four mice per group. ^∗^p < 0.05 and ^∗∗^p < 0.01 relative to the control *Akita* group; ^##^p < 0.01 relative to the *Akita*-*Tsc1*^fl/fl^ group.

Next, we studied whether stimulation of mTORC1 abrogates the renal beneficial effects of dapagliflozin in diabetes. We generated *Sglt2-Cre*;*Tsc1*^*fl/fl*^ *Akita* mice, in which RPTC mTORC1 activity was further increased, compared with control diabetic *Akita* mice (Figures 5A and 5B). Similar to *Tsc1* deletion in wild-type mice, this genetic manipulation in *Akita* mice also increased glucosuria and diuresis without affecting glycemia, along with decreased body weight and increased kidney/body weight ratio compared with *Tsc1*^*+/+*^ *Akita* mice (Figures S6A and S6E). In addition, the urinary excretion of calcium and phosphate, but not of sodium and uric acid, was increased in *RPTC-Tsc1*-KO *Akita* mice (Figures S6I–S6L), suggesting that mTORC1 may have a more general role in nutrient transport in RPTCs, as previously reported (Grahammer et al., 2017). Intriguingly, increased diuresis and glucosuria in *RPTC-Tsc1*-KO *Akita* mice completely prevented glomerular hypertrophy (compare Figures S6F–S6H and Figures 1C–1E). Twelve-week treatment with dapagliflozin failed to inhibit mTORC1 in *RPTC-Tsc1*-KO (*Sglt2Cre;Tsc1*^*fl/fl*^) *Akita* mice (Figures 5A and 5B), indicating that dapagliflozin acts upstream to the Tsc1/Tsc2 complex. Under these conditions, dapagliflozin did not further increase glucosuria and did not affect the glomerular and Bowman’s capsule areas (Figures S6E–S6H). Tubular dysfunction was increased in *RPTC-Tsc1*-KO *Akita* compared with control *Akita* mice, evident by increased cystatin-C, urinary KIM-1, and albuminuria (Figures 5C, 5E, and 5F). Treatment of *RPTC-Tsc1*-KO *Akita* mice with dapagliflozin partially decreased cystatin-C expression, but there was no effect on albuminuria, KIM-1 excretion, or fibrosis (collagen ΙΙΙ) (Figures 5C–5F).

Treatment with dapagliflozin did not affect BUN and serum creatinine in *RPTC-Tsc1* KO (Figures 5G and 5H). Creatinine clearance was somewhat higher in the *RPTC-Tsc1*-KO *Akita* than in control *Akita* mice (Figure 5I). *RPTC-Tsc1* KO had dual opposing effects on kidney function: on one hand, it promoted fibrogenesis, which is deleterious (Figure 4K); on the other hand, inhibition of sodium-glucose transport reduced hyperfiltration and may prevent glomerular injury (Figures S6F–S6H). Diabetes markedly increased fibrogenesis; it is plausible that under the pro-fibrogenic conditions of diabetes, the beneficial hemodynamic effects of *Tsc1* KO resulted in partial improvement of kidney function. Of note, in control *Akita* mice, dapagliflozin completely prevented the decline of creatinine clearance over time (Figure S1P). In contrast, in *RPTC-Tsc1*-KO *Akita* mice, dapagliflozin did not prevent the reduction of creatinine clearance, which remained ∼30% lower than in non-diabetic controls (compare Figure 5I and Figure S1P).

In summary, our findings show that stimulation of RPTC mTORC1 prevents the anti-fibrotic effects of SGLT2i and the preservation of renal function in diabetes.

### Inhibition of mTORC1 Activity in RPTCs Prevents Fibrosis along with Preservation of Kidney Function, Irrespective of Albuminuria

**Figure 6:**
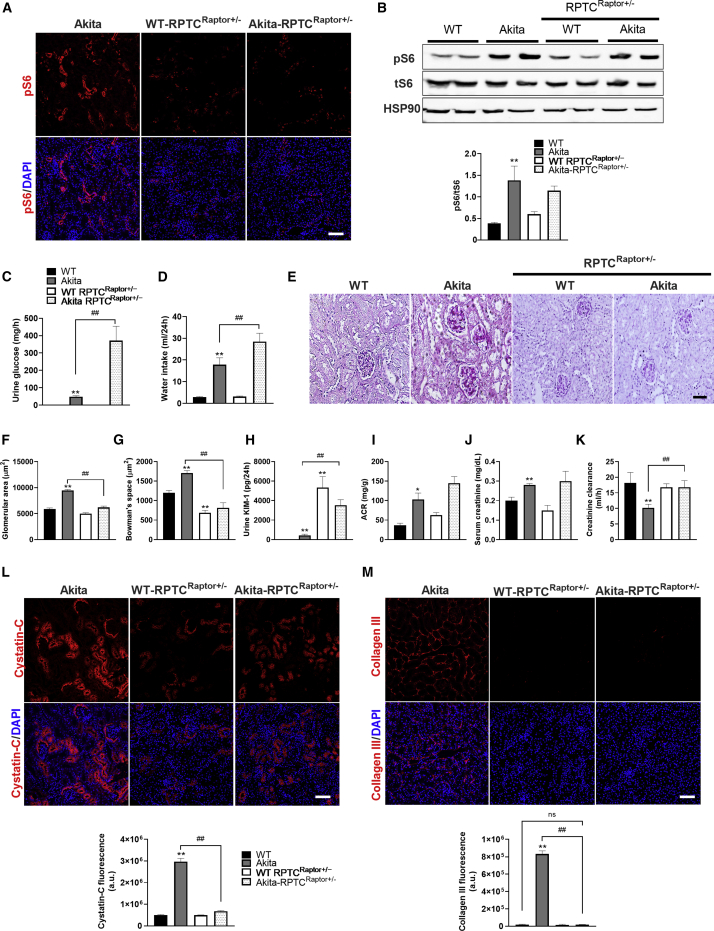
Partial Inhibition of mTORC1 by Heterozygous Raptor KO in RPTCs Prevents Fibrosis and Preserves Kidney Function RPTC-specific Raptor KO (*Sglt2Cre;Raptor*^*fl/+*^) in *Akita* and control mice was obtained by crossbreeding Raptor-floxed mice (*Raptor*^*fl/fl*^) with the *iL1-Sglt2-Cre* transgenic mice. Analyses were performed on 5-month-old mice. (A–M) mTORC1 activity based on immunofluorescence and western blotting for pS6 (A and B), (C) urine glucose, (D) 24 h water intake, (E–G) PAS staining (E) and quantifications of glomerular (F) and Bowman’s space (G) cross-sectional areas, (H and I) urine KIM-1 and ACR levels, (J) serum creatinine, (K) creatinine clearance, (L) immunofluorescence for cystatin-C, and (M) for collagen III. Scale bar, 50 μm. Data represent the mean ± SEM of four or five mice per group. ^∗^p < 0.05 and ^∗∗^p < 0.01 relative to the wild-type control group; ^##^p < 0.01 relative to the *Akita* control group.

To further corroborate the role of RPTC mTORC1 in DKD, we inhibited mTORC1 *in vivo* by conditional KO of the mTORC1 essential component *Raptor* in RPTCs. We generated *Sglt2-Cre*;*Raptor*^*fl/fl*^, *Sglt2-Cre*;*Raptor*^*fl/+*^ wild-type and *Akita* mice (Figure S7A). Previous studies showed that constitutive and inducible deletion of both *Raptor* alleles in renal tubular cells caused a Fanconi-like syndrome with glucosuria, phosphaturia, aminoaciduria, and albuminuria due to impairment of endocytosis and nutrient transport (Grahammer et al., 2017). Moreover, deletion of *Raptor* caused loss of tubular cells and increased the vulnerability of the kidney to ischemia and reperfusion injury (Grahammer et al., 2014). We therefore focused on mice with heterozygous *Raptor* KO and tested the effects of moderate inhibition of mTORC1 in control mice and in diabetic *Akita* mice in which RPTC mTORC1 is activated. We envisioned that partial, more physiological inhibition of mTORC1 should prevent its over-activation in diabetes and be beneficial rather than deleterious. Immunofluorescence showed that mTORC1 activity (S6 phosphorylation) was decreased in *RPTC-Raptor*^fl/+^ diabetic *Akita* mice and in *RPTC-Raptor*^fl/+^ wild-type mice compared with that of control *Akita RPTC-Raptor*^+/+^ (Figure 6A). Western blotting showed that pS6 protein level was modestly decreased in whole-kidney homogenates of *RPTC-Raptor*^fl/+^ *Akita* mice, indicating that the inhibition of mTORC1 activity in the heterozygous mice was indeed moderate (Figure 6B). *RPTC-Raptor* KO did not affect blood glucose, body weight, or kidney/body weight ratio in control and *Akita* mice (Figures S7B–S7D). Heterozygous deletion of *Raptor* in wild-type mice did not affect glucose excretion, water intake, or glomerular size. In contrast, heterozygous *Raptor* deletion in *Akita* mice markedly augmented glucosuria; this was associated with decreased glomerular size (Figures 6C–6G). *RPTC-Raptor* KO in *Akita* mice also increased the urinary excretion of sodium, phosphate, uric acid, calcium, albumin, and KIM-1 compared with *Akita* controls (Figures S7E–S7H; Figures 6H and 6I). These findings suggest that partial inhibition of mTORC1 impairs RPTC transport functions; this defect is further increased in diabetes.

Cystatin-C expression was decreased in RPTCs of *RPTC-Raptor*^fl/+^ *Akita* mice compared with *Akita* controls (Figure 6L). Strikingly, heterozygous *Raptor* KO completely prevented diabetes-induced fibrosis (Figure 6M) and the associated decline in creatinine clearance (Figure 6K). We conclude that moderate inhibition of RPTCs mTORC1 activity is sufficient to prevent interstitial fibrosis and the development of diabetic renal failure.

### Mechanisms of mTORC1-Induced Renal Fibrosis

**Figure 7:**
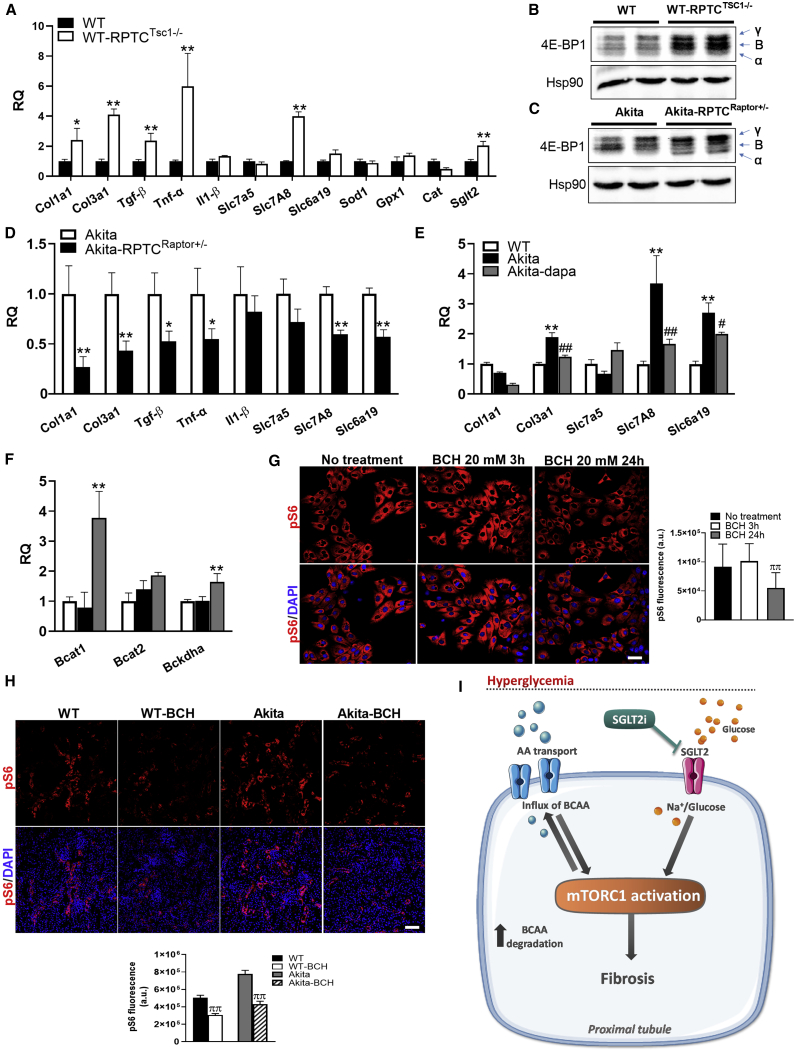
mTORC1 Regulation of Fibrogenesis, Amino Acid and Glucose Transport, Oxidative Stress, and Pro-inflammatory Genes (A) Gene expression in kidney cortex extracts of 5-month-old RPTC-specific Tsc1-KO (*Sglt2Cre;Tsc1*^*fl/fl*^) mice compared with controls. (B) 4E-BP1 phosphorylation. (C and D) 4E-BP1 phosphorylation (C) and gene expression (D) in kidney cortex extracts of 5-month-old Akita mice and RPTC-specific Raptor-KO (*Sglt2Cre;Raptor*^*fl/+*^) Akita mice. (E and F) Expression of collagen, amino acid transporters (E), and BCAA-degrading enzymes (F) in Akita mice treated with and without dapagliflozin for 3 months compared with wild-type (WT) controls. (G) LLC-PK1 cells treated with 20 mM BCH or left untreated for 3 and 24 h. mTORC1 activity was analyzed using pS6 immunostaining. Quantification of fluorescence intensity is shown. (H) Wild-type and Akita mice were intraperitoneally (i.p.) injected with BCH (2 μM/g body weight per day) for 5 days. mTORC1 activity was analyzed using immunostaining for pS6 and quantification of pS6 fluorescence intensity in RPTCs. (I) A mechanistic model of the effects of diabetes and of treatment with SGLT2i on the development and progression of DKD (see text for details). Scale bar, 50 μm. Data represent the mean ± SEM of three to five mice per group. ^∗^p < 0.05 and ^∗∗^p < 0.01 relative to the control wild-type or *Akita* groups; ^#^p < 0.05 and ^##^p < 0.01 relative to the Akita control group; ^ππ^p < 0.01 relative to the BCH untreated control group.

Our findings suggest that mTORC1 activity in RPTCs modulates fibrogenesis. To clarify the mechanisms involved, we compared the expression of genes regulating fibrogenesis, oxidative stress, inflammation, and senescence in the renal cortex of RPTC-*Tsc1*-KO and control mice. mTORC1 activation in RPTCs increased *Collagen 1*, *Collagen 3*, *Tgfβ*, *Tnfα*, and the neutral amino acid transporter *Slc7a8*, whereas the expression of other genes regulating inflammation and oxidative stress was unchanged (Figure 7A). In addition, *Tsc1* KO in RPTCs increased the expression of *Sglt2* (Figure 7A), further suggesting that mTORC1 regulates the glucose transport system in RPTCs. mTORC1 has been recently shown to regulate TGFβ-induced fibrogenesis through phosphorylation of eukaryotic translation initiation factor 4E (eIF4E)-binding protein 1 (4E-BP1) (Woodcock et al., 2019). In RPTC-*Tsc1*-KO mice, 4E-BP1 phosphorylation was indeed increased (Figure 7B), suggesting that the mTORC1-4E-BP1-TGFβ axis promotes renal fibrosis.

We then studied the effects of *Raptor* KO in RPTCs on 4E-BP1 activity and gene expression in the diabetic kidney. 4E-BP1 dephosphorylation was reflected in the shift from the highly phosphorylated γ- and β-bands to the nonphosphorylated α-band (Figure 7C), as previously reported (Ni et al., 2017). Consistent with the above paradigm, inhibiting mTORC1 in *Akita* mice decreased the expression of genes regulating fibrosis, inflammation, and amino acid transport, including *Collagen ΙΙΙ*, *Collagen Ι*, *Tgfβ*, *Tnfα*, and the amino acid transporters *Slc7a8* and *Slc6a19* (Figure 7D). Collectively, these findings suggest that mTORC1 activates a pro-fibrotic program, involving increased collagen gene expression and modulation of amino acid transport.

Finally, we compared gene expression in the renal cortex of diabetic *Akita* mice treated with or without dapagliflozin. *Collagen ΙΙΙ*, *Slc7a8*, and *Slc6a19* expression was increased in diabetic animals, whereas treatment with SGLT2i reversed these alterations (Figure 7E). In addition, treatment with dapagliflozin was also associated with increased expression of key enzymes involved in BCAA degradation (Figure 7F), which may contribute to SGLT2i inhibition of mTORC1. We further studied the role of amino acid transport in the regulation of mTORC1 in RPTCs. Treatment of LLC-PK1 cells with the pan-L-type amino acid transporter inhibitor 2-amino-2-norbornaecarboxylic acid (BCH) inhibited mTORC1 activity (Figure 7G). In addition, BCH injection to WT mice and *Akita* mice for 5 days decreased mTORC1 activity in RPTCs *in vivo* (Figure 7H).

Of note, the expression of genes involved in mitochondrial biogenesis, oxidative stress, and senescence, as well as AMPK activity and HIF1α protein level and localization, remained unchanged in diabetic kidneys (Figures S7I–S7K), suggesting that mTORC1 stimulates fibrogenesis independent of these pathways.

We conclude that inhibition of amino acid transport and probably increased BCAA degradation along with decreased glucose transport explain the marked inhibition by SGLT2i of mTORC1 in RPTCs, thereby preventing fibrogenesis.

## Discussion

We show here that mTORC1 activity in RPTCs plays a key role in the pathophysiology of DKD and in mediating the renal-protective effects of SGLT2i. mTORC1 activity in RPTCs was increased in diabetes, whereas treatment with the SGLT2i dapagliflozin inhibited mTORC1 along with inhibition of tubular injury and fibrosis (Figure 7I). Strikingly, stimulation of mTORC1 in RPTCs by deleting *Tsc1* in normoglycemic mice induced tubular hypertrophy, tubular injury, and peritubular fibrosis with subsequent development of albuminuria and renal failure, thus mimicking DKD in the absence of hyperglycemia. Moreover, *Tsc1* deletion in RPTCs of diabetic animals prevented the inhibition of mTORC1 by dapagliflozin; this abolished the renal-protective effects of SGLT2i in diabetes. Importantly, moderate reduction of mTORC1 activity by conditional, heterozygous deletion of *Raptor* in RPTCs completely prevented fibrosis and preserved renal function. The importance of mTORC1 in DKD pathophysiology is amply emphasized by the fact that moderate changes in RPTC mTORC1 activity were sufficient to exert robust effects on fibrosis: deletion of a single *Tsc1* allele induced fibrogenesis, whereas partial deletion of *Raptor* prevented diabetes-induced renal fibrosis.

Fibrosis is the driving force of renal failure in diabetes, as well as in other kidney diseases. mTORC1 signaling to 4E-BP1 has been shown to mediate TGF-β-stimulated collagen synthesis in different tissues and thus represents a critical signaling node during fibrogenesis (Woodcock et al., 2019). Consistently, we found that mTORC1 stimulates 4E-BP1 phosphorylation and increases the expression of TGF-β, collagen, and certain amino acid transporters, including Slc6a19 and Slc7a8. Slc6a19 (B^0^AT1) is localized to the apical membrane of RPTCs and absorbs neutral amino acids from the lumen with high affinity to BCAA (Bröer et al., 2004). Slc7a8 (LAT2) is a system L transporter isoform, which forms a heterodimeric amino acid transporter with the 4F2 heavy chain (4F2hc) (Rossier et al., 1999). The SLC7A8/4F2hc complex is localized to the basolateral membrane of RPTCs and is involved in cysteine efflux in exchange of other neutral amino acids (Fernández et al., 2003). These alterations may promote protein synthesis including collagen, thereby promoting fibrosis.

A previous phosphoproteomic analysis of RPTC-Raptor KO showed reduced phosphorylation of several amino acid transporters and of SGLT2 along with aminoaciduria and glucosuria (Grahammer et al., 2017), suggesting that mTORC1 activity regulates tubular transport of nutrients and electrolytes (Grahammer et al., 2014, 2017). We found that tight regulation of mTORC1 activity seems to be essential for physiological tubular transport functions: both mTORC1 activation (*Tsc1* deletion) and inhibition (*Raptor* deletion) induced glucosuria and increased urinary KIM-1 and albumin excretion. Intriguingly, while deletion of *Raptor* in RPTCs in diabetic mice markedly increased glucosuria, albuminuria (in the absence of glomerular hypertrophy), and urinary KIM-1 beyond the levels observed in non-diabetic mice, suggesting tubular dysfunction, still inhibition of mTORC1 completely prevented fibrosis and consequently the decline in creatinine clearance. Thus, mTORC1 effects on tubular transport, albuminuria, and kidney function in diabetes can be dissociated.

mTORC1 seems to function as a double-edged sword: on one hand, it is vital for organ morphogenesis and function, and on the other hand, its sustained activation may lead to tissue dysfunction (Ardestani et al., 2018). This duality is demonstrated in the pleiotropic effects of mTORC1 in the kidney. mTORC1 is essential for glomerular development and for the integrity of the filtration barrier, as well as for the maintenance of tubular cells and transport functions (Gödel et al., 2011; Inoki et al., 2011). However, sustained activation of mTORC1 in RPTCs promotes tubular dysfunction and fibrosis, leading to renal failure. Multiple mechanisms could be involved in mTORC1-induced tubular injury, including increased apoptosis and/or dedifferentiation. We found no evidence of increased RPTCs apoptosis or of epithelial-mesenchymal transition of RPTCs in diabetes (Figures S3A and S3B); therefore, we believe that the deleterious effects of mTORC1 in diabetes are not mediated via effects on RPTC turnover and/or differentiation. Intriguingly, in diabetic animals, there were no significant changes in the expression of genes involved in oxidative stress and inflammation. Tubular hypertrophy along with accumulation of peritubular fibrotic tissue could lead to tubular cell hypoxia, especially in RPTCs, which are metabolically highly active and rely on intense oxidative phosphorylation. We found no evidence for HIF1α stabilization or nuclear translocation in diabetic animals treated with or without SGLT2i. Although we cannot exclude that these pathways are involved in the pathophysiology of DKD and in mediating the beneficial effects of SGLT2i, we believe that our findings suggest that mTORC1 is a direct regulator of fibrogenesis. Tubulointerstitial fibrosis has a robust effect on kidney function, probably explaining the strong renal-protective effects of SGLT2i even in patients with advanced kidney disease.

Previous studies showed that in diabetes, mTORC1 activity is increased in podocytes, leading to glomerular cell hypertrophy, foot process effacement, and eventually detachment from the glomerular basement membrane (Gödel et al., 2011; Inoki et al., 2011). These studies along with our findings suggest that dysregulated mTORC1 activity has a general role in mediating the deleterious effects of the diabetic environment on kidney morphology and function. The accepted paradigm is that glomerular alterations are an early event, preceding tubule-interstitial damage in DKD. Our findings emphasize the importance of “tubulopathy” in the pathophysiology of DKD: tubular hypertrophy and injury preceded glomerular hypertrophy, and early treatment with dapagliflozin prior to development of glomerular hypertrophy decreased tubular dysfunction. Moreover, *Tsc1* deletion in RPTCs of *Akita* mice was associated with decreased glomerular size, probably because of enhanced sodium delivery to the distal part of the nephron, but still was ineffective in preventing the decline of renal function in diabetes by treatment with SGLT2i.

In diabetes, mTORC1 can be activated by nutrient overload elicited by hyperglycemia and probably by other nutrients, such as BCAA that are increased in the sera of subjects with metabolic syndrome and obesity (Batch et al., 2013). RPTCs might be particularly vulnerable to the nutrient overload because of their unique role in nutrient (i.e., glucose and amino acid) transport from the kidney lumen to the blood. Normally, ∼180 g glucose and 2 g amino acids are filtered daily in the glomerulus, the majority undergoing reuptake by RPTCs via SGLT2 and amino acid transporters. Thus, these cells are extensively exposed to glucose and amino acids, which probably explains their high basal mTORC1 activity, which in turn promotes mitochondrial biogenesis and energy production. In diabetes, the workload on RPTCs is further increased because of higher glucose filtration in the glomerulus followed by its reuptake by RPTCs; this, along with increased exposure to BCAA, can further stimulate mTORC1. This scenario is supported by our *in vitro* studies showing that incubation of RPTCs at high glucose increased basal mitochondrial respiration together with stimulation of mTORC1. Treatment with SGLT2i decreased glycolysis, mitochondrial respiration, and mTORC1 activity, indicating that treatment of diabetes with SGLT2i decreases the workload of RPTCs by reducing the flux of glucose through the cell and its metabolism. SGLT2i also decreased the expression of neutral amino acid transporters, including BCAA, and increased the expression of BCAA degrading enzymes. The dual inhibition of glucose and BCAA uptake by SGLT2i may explain its robust inhibition of mTORC1 in RPTCs.

### Study Limitations

The studies were performed in a rodent model of DKD, and it remains unclear to what extent the SGLT2i mode of action in this model recapitulates that in human DKD. Nevertheless, the *Akita* mouse recapitulates many of the features of DKD in humans, including glomerular hypertrophy, mesangial expansion (in aged diabetic mice), albuminuria, and worsening of kidney function over time (Chang et al., 2012; Kitada et al., 2016); thus we believe the *Akita* mouse is an adequate model for studying the mechanisms of DKD. Furthermore, mTORC1 was similarly activated in additional models of T1D (STZ-induced diabetes) and T2D (db/db mice); thus we believe that our findings can be generalized to other forms of diabetes and probably also to the human disease. There is compelling evidence showing that tubule-interstitial alterations are common in diabetes and that SGLT2i modulates molecular signatures associated with inflammation, adhesion molecule turnover, and fibrosis in humans (Heerspink et al., 2019). The pathophysiology of human DKD is heterogeneous, but we believe that the above-described mechanisms play a role at least in part of patients with DKD. Further studies are required to confirm that RPTC mTORC1 mediates renal fibrosis in human subjects and to identify those who will benefit from treatment with SGLT2i.

### Clinical Implications

Our findings have important therapeutic implications for the prevention and treatment of DKD and for the mechanism(s) of action of SGLT2i. We suggest that the main mechanism of kidney protection by SGLT2i is in fact reduction of glucose and BCAA metabolism in the RPTCs, which is the site of action of SGLT2i, independent of the systemic metabolic effects. Importantly, it has been recently formally announced that in the Dapagliflozin and Prevention of Adverse Outcomes in Chronic Kidney Disease (DAPA-CKD) phase III trial (NCT03036150), dapagliflozin showed overwhelming efficacy in preventing adverse renal outcomes, irrespective of the presence of diabetes (unpublished data), further suggesting that the protective effects of SGLT2i are mediated via direct renal effects, rather than by modulating the diabetic metabolic milieu. RPTCs seem to play a key role in the pathophysiology of DKD; therefore modulation of nutrient transport and consequently the activity of key signaling pathways such as mTORC1 that are highly active in these cells may have robust effects on kidney dysfunction in diabetes.

We finally propose that RPTC mTORC1 be regarded as a therapeutic target for DKD and probably other kidney diseases. Unraveling the metabolic pathways leading to mTORC1 activation by glucose and BCAA in RPTCs may facilitate the development of new therapeutic approaches for DKD and probably also other kidney disease.

## STAR★Methods

### Key Resources Table

**Table:** 

REAGENT or RESOURCE	SOURCE	IDENTIFIER
**Antibodies**		

Rabbit anti pS6	Cell Signaling Technology	Cat#5364S; RRID:AB_10694233
Rabbit anti Cystatin-C	Abcam	Cat#ab109508; RRID:AB_10888303
Rabbit anti collagen III	Abcam	Cat#ab7778; RRID:AB_306066
Rabbit anti TNFα	Abcam	Cat#ab6671; RRID:AB_305641
Goat anti-GFP	Abcam	Cat#ab6673; RRID:AB_305643
Mouse anti SMA	Abcam	Cat#ab7817; RRID:AB_262054
Rabbit anti-ZO1	Abcam	Cat#ab216880
Rabbit anti E-cadherin	Abcam	Cat#ab15148; RRID:AB_301693
Rabbit anti-Ki67	Thermo Scientific	Cat#MA5-14520; RRID:AB_10979488
Rabbit anti-pAKT S473	Cell Signaling Technology	Cat#4060S; RRID:AB_2315049
Rabbit anti HIF-1 alpha	Abcam	Cat#ab82832; RRID:AB_1860665
Rabbit anti Ts6	Cell Signaling Technology	Cat#2217S; RRID:AB_331355
Mouse anti HSP90	Abcam	Cat#ab13495; RRID:AB_1269122

**Chemicals, Peptides, and Recombinant Proteins**		

Dapagliflozin (Forxiga)	AstraZeneca	N/A
Dapagliflozin	Cayman Chemical	Cat# 11574
Insulin degludec	Novo Nordisk	N/A
2-amino-2-norbornaecarboxylic acid (BCH)	Sigma-Aldrich	Cat#A7902
TRIzol	Bio-Lab	Cat# 959758027100
Periodic Acid–Schiff	Sigma-Aldrich	Cat#395B-1KT
Rapamycin	Cell Signaling	Cat#9904
Torin-1	Cell Signaling	Cat#14379
Clarity Western ECL Blotting Substrate	Bio-Rad	Cat#1705061
FastStart SYBR-Green Master	Applied Biosystems	Cat#4385610

**Critical Commercial Assays**		

β-Hydroxybutyrate colorimetric assay	Cayman Chemical	Cat#700190
Insulin ELISA kit	Crystal Chem	Cat#90080
BCAA kit	BioVision	Cat#K564
Albumin kit	Bethyl Laboratories	Cat#E99-134
KIM-1 kit	R&D Systems	Cat#MKM100
XF cell mito stress test kit	Agilent Technologies	Cat#103015-100
cDNA synthesis kit	Applied Biosystems	Cat#4368814

**Experimental Models: Cell Lines**		

LLC-PK1	ATCC	Cat#CL-101
HK-2	ATCC	Cat#CRL-2190

**Experimental Models: Organisms/Strains**		

Akita	Jackson Laboratories	Cat# 003548
Sglt2Cre;Tsc1^fl/fl^	This paper	N/A
Sglt2Cre;Raptor^fl/+^	This paper	N/A
Sglt2-Cre; Rosa26-YFP	This paper	N/A

**Oligonucleotides**		

RT-PCR primers	See STAR Methods for all the primer sequences	N/A

**Software and Algorithms**		

ImageJ	ImageJ Software	https://imagej.nih.gov/ij/
GraphPad Prism	GraphPad Software	https://www.graphpad.com
NIS-Elements software	NIS-Elements software	Nikon instruments Inc.

### Resource Availability

#### Lead Contact

Further information and requests for resources and reagents should be directed to and will be fulfilled by the Lead Contact, Prof. Gil Leibowitz (GLEIB@hadassah.org.il).

#### Materials Availability

All unique reagents generated in this study are available from the Lead Contact.

#### Data and Code Availability

This study did not result in any datasets or custom code.

### Experimental Model and Subject Details

#### Animals

The experimental protocol was approved by the Institutional Animal Care and Use Committee of the Hebrew University of Jerusalem. To study the impact of SGLT2 inhibition on DKD, 8-week old male *Akita* (*Ins*2^WT/C96Y^) (Jackson Laboratories) diabetic mice were treated with or without SGLT2i (dapagliflozin (Forxiga), AstraZeneca); 10 mg/kg/day in drinking water) for 12 weeks or 5 days. When specified, mice were given a subcutaneous injection of insulin (4-6 U/day, degludec (Tregludec), Novo Nordisk). Body weight and blood glucose were monitored weekly. Before euthanizing, mice were placed in the CCS2000 Chiller System for 24 h urine collection (Hatteras Instruments). After euthanasia, blood was collected, the kidneys were removed and weighed, and either snap-frozen or fixed in buffered 4% paraformaldehyde and embedded in paraffin.

RPTC-specific *Tsc1* knockout mice (*Sglt2Cre;Tsc1*^fl/fl^ and *Sglt2Cre; Tsc1*^fl/+^) were generated by crossbreeding TSC1-floxed mice (*Tsc1*^*fl/fl*^) with *iL1-Sglt2-Cre* transgenic mice (Rubera et al., 2004) which expresses Cre recombinase in the brush border membrane of the S1 segment of the proximal tubule. Mice lacking Raptor in RPTCs (*Sglt2Cre;Raptor*^*fl/+*^) were generated by crossbreeding raptor-floxed mice (*Raptor*^*fl/fl*^; Jackson Laboratories) with the *iL1-Sglt2-Cre* transgenic mice. RPTC-specific labeling was achieved by crossing *Rosa26*^*stopYFP*^ (Srinivas et al., 2001) with the *iL1-Sglt2-Cre* transgenic mice. All transgenic mice were generated on the background of the *Akita* and WT mice.

Assessment of the effects amino acid transport on mTORC1 activity was performed by IP injection of the L-type amino acid transporter inhibitor 2-amino-2-norbornaecarboxylic acid (BCH).

#### Cell lines

The RPTC cell lines LLC-PK1 (pig) (Hull et al., 1976) and HK-2 (human) (Ryan et al., 1994) were used for *in vitro* studies on glucose and dapagliflozin effects on mTORC1 activity. LLC-PK1 and HK-2 cells were cultured in DMEM supplemented with 10% fetal bovine serum and 100 IU/mL penicillin/streptomycin (Biological Industries) at 37 °C in a humid atmosphere with 5% CO_2_.

### Method Details

#### Blood and urine biochemistry

BUN, urine glucose and urine and serum creatinine were determined using Cobas C-111 chemistry analyzer (Roche). β-Hydroxybutyrate was determined using a colorimetric assay (Cayman). Plasma insulin was determined using an ELISA kit (Crystal Chem). Plasma BCAA were determined using a colorimetric kit (BioVision). Urine albumin and KIM-1 were measured by ELISA kits (Bethyl Laboratories and R&D Systems). Urinary sodium, calcium, phosphate, and uric-acid concentrations were determined by colorimetric methods (Lehmann).

#### Histopathological analysis

Paraffin-embedded kidney sections were stained with Periodic Acid–Schiff (PAS, Sigma-Aldrich) followed by hematoxylin staining. Kidney images were taken from 10 random 20x fields from each animal. Glomerular and Bowman’s space areas and the mesangial area were quantified in 40 randomly chosen glomeruli using the NIS-Elements software (Nikon instruments Inc.).

#### Immunofluorescence staining

Paraffin sections were rehydrated and antigen retrieval was performed using citrate buffer (pH 6). The following antibodies were used: rabbit anti pS6 (5364S, Cell Signaling), rabbit anti Cystatin-C (ab109508, Abcam), rabbit anti collagen III (ab7778, Abcam), rabbit anti TNFα (ab6671, Abcam), goat anti-GFP (ab6673, Abcam), mouse anti SMA (ab7817, Abcam), rabbit anti-ZO1 (ab216880, Abcam), rabbit anti E-cadherin (ab15148, Abcam), rabbit anti-Ki67 1:200 (Thermo Scientific), rabbit anti-pAKT S473 (4060S, Cell Signaling), rabbit anti HIF-1 alpha (ab82832, Abcam). Cell nuclei were visualized with DAPI staining. Secondary antibodies were all from Jackson ImmunoResearch Laboratories. Digital images were obtained with a Nikon A1R confocal microscope. Fluorescence was quantified using the ImageJ software (NIH, Bethesda, MD).

#### Cell culture

LLC-PK1 and HK-2 cells were cultured in DMEM supplemented with 10% fetal bovine serum and 100 IU/mL penicillin/streptomycin (Biological Industries) at 37 °C in a humid atmosphere with 5% CO_2_. For studying S6 phosphorylation, cells were starved in serum-free DMEM for 1 h and then exposed to either 5 mM or 30 mM D-glucose (low and high glucose, respectively) in the presence or absence of SGLT2i (dapagliflozin; 5 μM, Cayman Chemical) for 0.5 h. For immunofluorescence, cells were exposed to either 5 mM or 30 mM D-glucose in the presence or absence of SGLT2i (5 μM) for 48 h. Rapamycin and Torin-1 (Cell Signaling) were added to the medium at a concentration of 100 and 250 nM, respectively. For inhibiting system L amino acid transporters, cells were treated with 20 mM BCH (Sigma-Aldrich) for 24 h.

For immunofluorescence, cells were grown on glass coverslips and fixed with 4% paraformaldehyde. After permeabilization and blocking, cells were incubated with anti-pS6 antibodies (5364S, Cell Signaling) followed by incubation with Cy3-conjugated anti-rabbit secondary antibodies (Jackson ImmunoResearch Laboratories). Cells were mounted on slides and examined with a Nikon A1R confocal microscope.

#### Seahorse analysis

The metabolic profile of cultured LLC-PK1 cells was assessed using the XFe96 Seahorse analyzer and XF cell mito stress test kit (Agilent Technologies). Cells were cultured in 96-well assay plates at a density of 2 × 10^3^ cells/well in complete growth medium containing 5 mM or 30 mM glucose with or without SGLT2i for 24 h. Before starting the assay, the growth medium was changed to unbuffered DMEM, pH 7.4, and cells were incubated at 37°C without CO_2_ for 1 h for equilibration. Oxygen consumption rate (OCR) and extracellular acidification rate (ECAR) were measured simultaneously in repeated cycles to obtain basal rates. After baseline measurements, OCR and maximal ECAR were measured after the injection of oligomycin (1.5 μM). Subsequently, carbonylcyanide-4-(trifluoromethoxy) phenylhydrazone (FCCP, 1 μM) was injected and the maximal OCR measured. Non-mitochondrial oxygen consumption was measured after the injection of rotenone and antimycin A (0.5 μM each). OCR and ECAR rates were normalized to cell number as was indicated by Hoechst staining.

#### Western blotting

Kidney or cell homogenates were prepared in RIPA buffer (25mM Tris-HCl pH 7.6, 150 mM NaCl, 1% NP-40, 1% sodium deoxycholate, 0.1% SDS). Samples were resolved by 12% SDS-PAGE and transferred to nitrocellulose membranes. After blocking, blots were incubated overnight with rabbit anti-pS6 (5364S, Cell Signaling), tS6 (2217S, Cell Signaling Technology) and HSP90 (ab13495, Abcam) antibodies at 4°C. Anti-rabbit horseradish peroxidase (HRP)-conjugated secondary antibodies (Jackson ImmunoResearch Laboratories) were used for 1 h at room temperature and followed by chemiluminescence detection using Clarity Western ECL Blotting Substrate (Bio-Rad). Relative band intensities were quantified by the ImageJ software.

#### Real-time RT-PCR

Total RNA was extracted from renal cortex using TRIzol (Bio-Lab), and cDNA was synthesized using 2 μg of RNA by reverse transcription (Applied Biosystems). Real-time PCR (RT-PCR) was performed with FastStart SYBR-Green Master (Applied Biosystems) using an ABI PRISM 7000 Sequence Detection System (Applied Biosystems). The mRNA levels of all genes were normalized to GAPDH. qPCR primers used in this study were as follow:

*Col1a1* F: ACAAGGTGACAGAGGCATAAA,
*Col1a1* R: ACCAGGAGAACCAGGAGAA;
*Col3a1* F: GGCTGCAAGATGGATGCTATAA,
*Col3a1* R: GAATCTGTCCACCAGTGCTTAC;
*Tgf-β* F: CTGAACCAAGGAGACGGAATAC,
*Tgf-β* R: GGGCTGATCCCGTTGATTT;
*Tnf-α* F: GCCTCCCTCTCATCAGTTCTAT,
*Tnf-α* R: CACTTGGTGGTTTGCTACGA;
*Il1-β* F: CCACCTCAATGGACAGAATATCA,
*Il1-β* R: CCCAAGGCCACAGGTATTT;
*Slc7a5* F: CCTACGGAGGATGGAACTATCT,
*Slc7a5* R: TGACAATGGGCAAGGAGATG;
*Slc7a8* F: GGTGGCTGGAACTTCCTTAAT,
*Slc7a8* R: CAGTGGGATGGAGATGAAGATG;
*Slc6a19* F: GGAGTGTGCTGTATGTGTGTAT,
*Slc6a19* R: TCAAGCCACGGATGAGAAAG;
*Sod1* F: GGTTCCACGTCCATCAGTATG,
*Sod1* R: GTCTCCAACATGCCTCTCTTC;
*Gpx1* F: CACCAGGAGAATGGCAAGAA,
*Gpx1* R: CATTCACTTCGCACTTCTCAAAC;
*Cat* F: GATGGTAACTGGGATCTTGTGG,
*Cat* R: GTGGGTTTCTCTTCTGGCTATG;
*Sglt1* F: GTGTACGGATCAGGTCATTGT,
*Sglt1* R: CATGGGCAGTAGCTTCAGATAG;
*Sglt2* F: CATTCAGTCTGTCTCCAGCTATC,
*Sglt2* R: GAAGGCTCCCTTCTCATTAACA;
*Bcat1* F: GTCTGCCCAGTCTCTGATATTC,
*Bcat1* R: ACTCTCCACCCTTCCATACT;
*Bcat2* F: GCTGATGGTGGAGTGGAATAA,
*Bcat2* R: CTCAAAGAGCTGCAGAGAGTAG;
*Nrf1* F: CTGAACACATGGCTACCATAGA,
*Nrf1* R: GGGAGTCTTCATCAGCACTTAG;
*Tfam* F: GGAATGTGGAGCGTGCTAAA,
*Tfam* R: TCGGAATACAGACAAGACTGATAGA;
*Pgc1α* F: GACACGAGGAAAGGAAGACTAAA,
*Pgc1α* R: GTCTTGGAGCTCCTGTGATATG;
*IL6* F: TTTCCTCTGGTCTTCTGGAGTA,
*IL6* R: CTCTGAAGGACTCTGGCTTTG;
*p16* F: GCAGATCCACAGCGATATCCA,
*p16* R: AACAGGTCGGACATCACCAG;
*p21* F: GTCTTGCACTCTGGTGTCTG,
*p21* R: GATAGAAATCTGTCAGGCTGGTC;
*GAPDH* F: CCCTTGAGCTAGGACTGGATAA,
*GAPDH* R: GGGCTGCAGTCCGTATTTATAG.

### Quantification and Statistical analysis

Quantification methods are described in the Method Details and in figure legends. Data are expressed as mean ± SEM. Unpaired two-tailed Student’s t test was used to determine differences between groups. Results in multiple groups and time-dependent variables were compared by ANOVA followed by Bonferroni test (GraphPad Prism 8.0.2). Differences were considered to be statistically significant at p < 0.05.

## Supplemental Information

Document S1. Figures S1–S7

Document S2. Article plus Supplemental Information
