# Supplementary material for: Proximal Tubule mTORC1 Is a Central Player in the Pathophysiology of Diabetic Nephropathy and Its Correction by SGLT2 Inhibitors
Source: Cell Rep. 2020 Jul 28;32(4):107954. doi: 10.1016/j.celrep.2020.107954 (PMC7397516; doi:10.1016/j.celrep.2020.107954)
Supplement: Document S1. Figures S1–S7 [file mmc1.pdf]

Cell Reports, Volume *32*

Supplemental Information

Proximal Tubule mTORC1 Is a Central Player in the Pathophysiology of Diabetic Nephropathy and Its Correction by SGLT2 Inhibitors

Aviram Kogot-Levin, Liad Hinden, Yael Riahi, Tal Israeli, Boaz Tirosh, Erol Cerasi, Ernesto Bernal Mizrachi, Joseph Tam, Ofri Mosenzon, and Gil Leibowitz

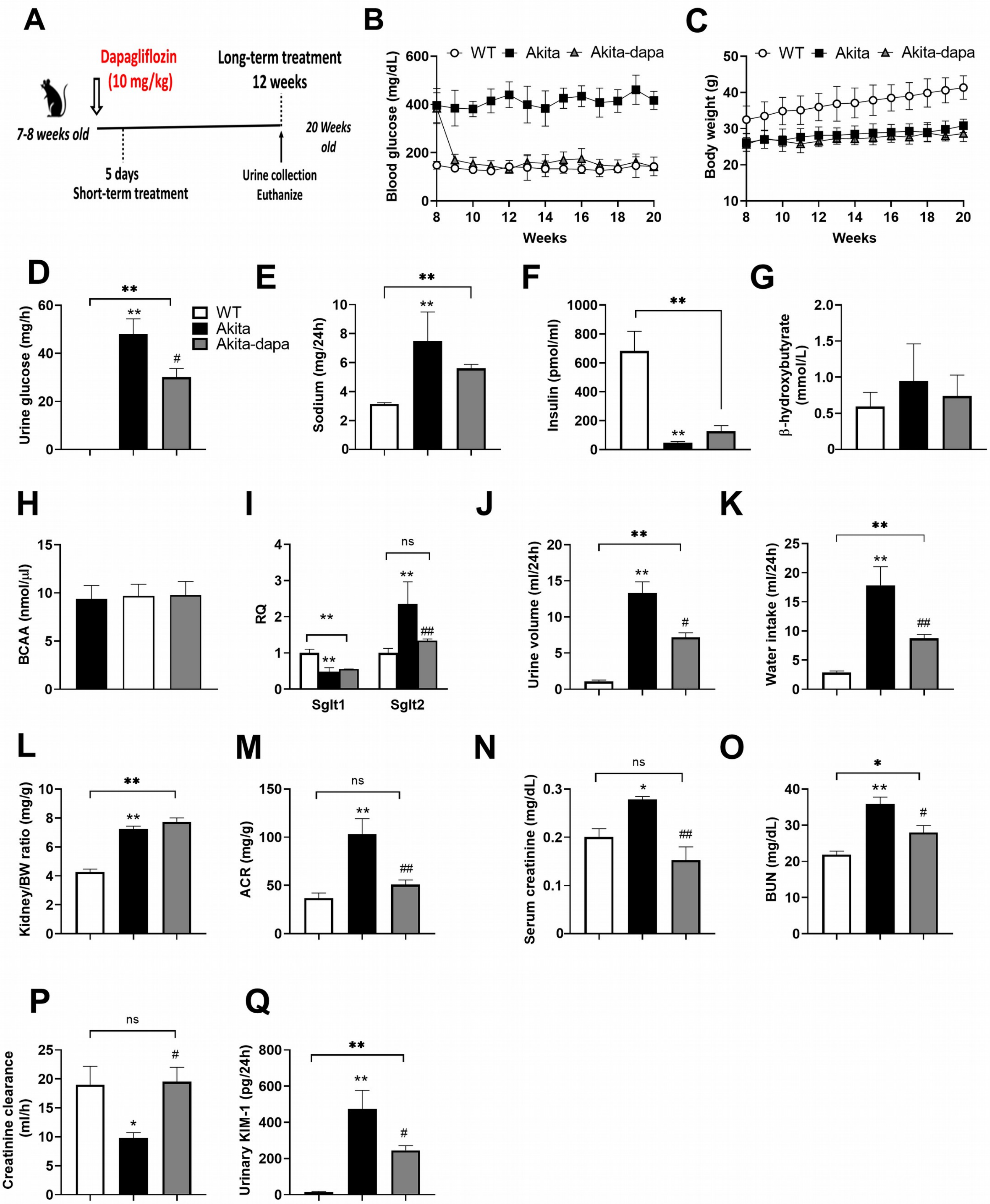
A
Dapagliflozin (10 mg/kg)
Long-term treatment
12 weeks
7-8 weeks old
20 Weeks old
5 days
Short-term treatment
Urine collection
Euthanize
B
WT
Akita
Akita-dapa
Blood glucose (mg/dL)
Weeks
C
Body weight (g)
D
Urine glucose (mg/h)
E
Sodium (mg/24h)
F
Insulin (pmol/ml)
G
β-hydroxybutyrate (mmol/L)
H
BCAA (nmol/μl)
I
RQ
Sglt1
Sglt2
ns
J
Urine volume (ml/24h)
K
Water intake (ml/24h)
L
Kidney/BW ratio (mg/g)
M
ACR (mg/g)
N
Serum creatinine (mg/dL)
O
BUN (mg/dL)
P
Creatinine clearance (ml/h)
Q
Urinary KIM-1 (pg/24h)

Figure S1. SGLT2i effects on glycemia, body weight and kidney function in Akita mice. Related to figure 1. (A) Schematic drawing of the experimental design; 7-8-week old male diabetic Akita (Ins2WT/C96Y) mice were treated with or without SGLT2i (dapagliflozin; 10 mg/kg/day in drinking water) for 12 weeks. (B) Fed blood glucose levels; (C) body weight (BW); (D-E) urine excretion of glucose and sodium; (F) serum insulin; (G) serum β-hydroxybutyrate; (H) serum BCAA; (I) mRNA expression levels of sglt1 and sglt2 in kidney cortex; (J-K) 24-h urine volume and water intake; (L) kidney/body weight ratio; (M) albuminuria, expressed as urine albumin-tocreatinine ratio (ACR); (N-P) kidney function assessed by serum creatinine, blood urea nitrogen (BUN) and creatinine clearance; (Q) urine KIM-1 levels. Data represent the mean ± SEM of 6-8 mice per group. *P<0.05, **P<0.01 relative to the wildtype control group; $^{\#}$P<0.05, $^{\#\#}$P<0.05 relative to the untreated Akita mice group; ns – non significant.

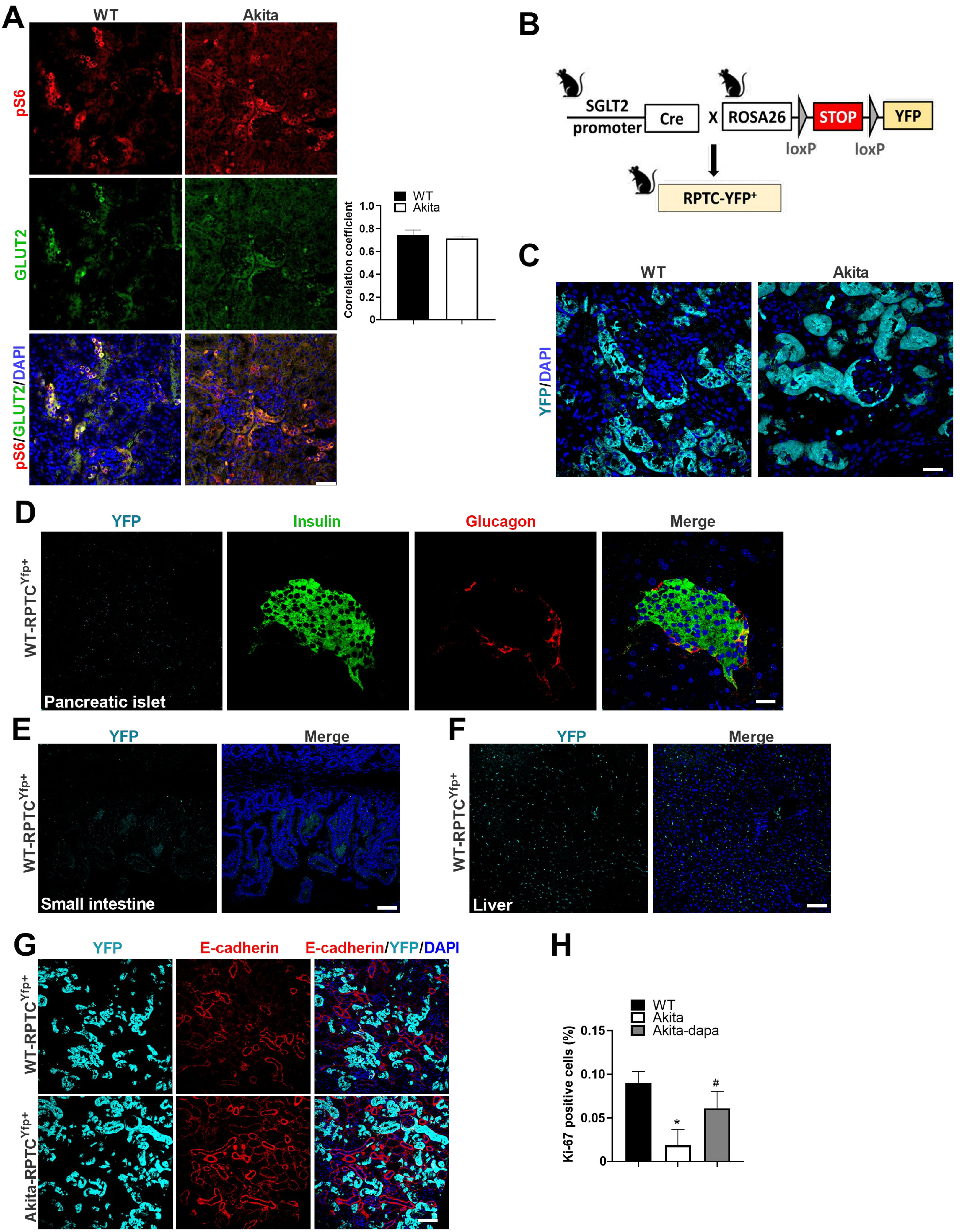
A
WT
Akita
pS6
GLUT2
pS6/GLUT2/DAPI
Correlation coefficient
WT
Akita
1.0
0.8
0.6
0.4
0.2
0
B
SGLT2
promoter
Cre
X
ROSA26
STOP
YFP
loxP
loxP
RPTC-YFP+
C
WT
Akita
YFP/DAPI
D
YFP
Insulin
Glucagon
Merge
WT-RPTCYfp+
Pancreatic islet
E
YFP
Merge
WT-RPTCYfp+
Small intestine
F
YFP
Merge
WT-RPTCYfp+
Liver
G
YFP
E-cadherin
E-cadherin/YFP/DAPI
WT-RPTCYfp+
Akita-RPTCYfp+
H
WT
Akita
Akita-dapa
Ki-67 positive cells (%)
0.15
0.10
0.05
0
*
#

Figure S2. **Assessment of mTORC1 activity in RPTCs and lineage tracing of RPTCs. Related to Figure 2.** (A) Immunofluorescence staining for pS6 and GLUT2 on renal sections of 8-week old wildtype and *Akita* mice. (B) Schematic diagram of the Cre mediated recombination strategy for generation of *RPTC-Rosa26-YFP+* reporter mice. (C) Immunofluorescence staining for YFP in lineage-traced (*RPTC-Rosa26*$^{YFP+}$) wildtype and *Akita* mice. (D) Immunostaining for YFP, insulin and glucagon on pancreatic sections of lineage-traced *Ssglt2-cre;Rosa26-*$^{YFP+}$ reporter mice. (E-F) YFP expression in small intestine (E) and liver (F). (G) Staining for YFP and distal tubule marker E-cadherin. (H) Assessment of RPTCs proliferation by quantification of the proliferation marker Ki67. The percentages of Ki67+/YFP+ cells are given. Scale bar, 50 μm. Data represent the mean ± SEM of 4 mice per group. *P<0.05 relative to the wild-type control group; $^{\#}$P<0.05 relative to the untreated *Akita* group.

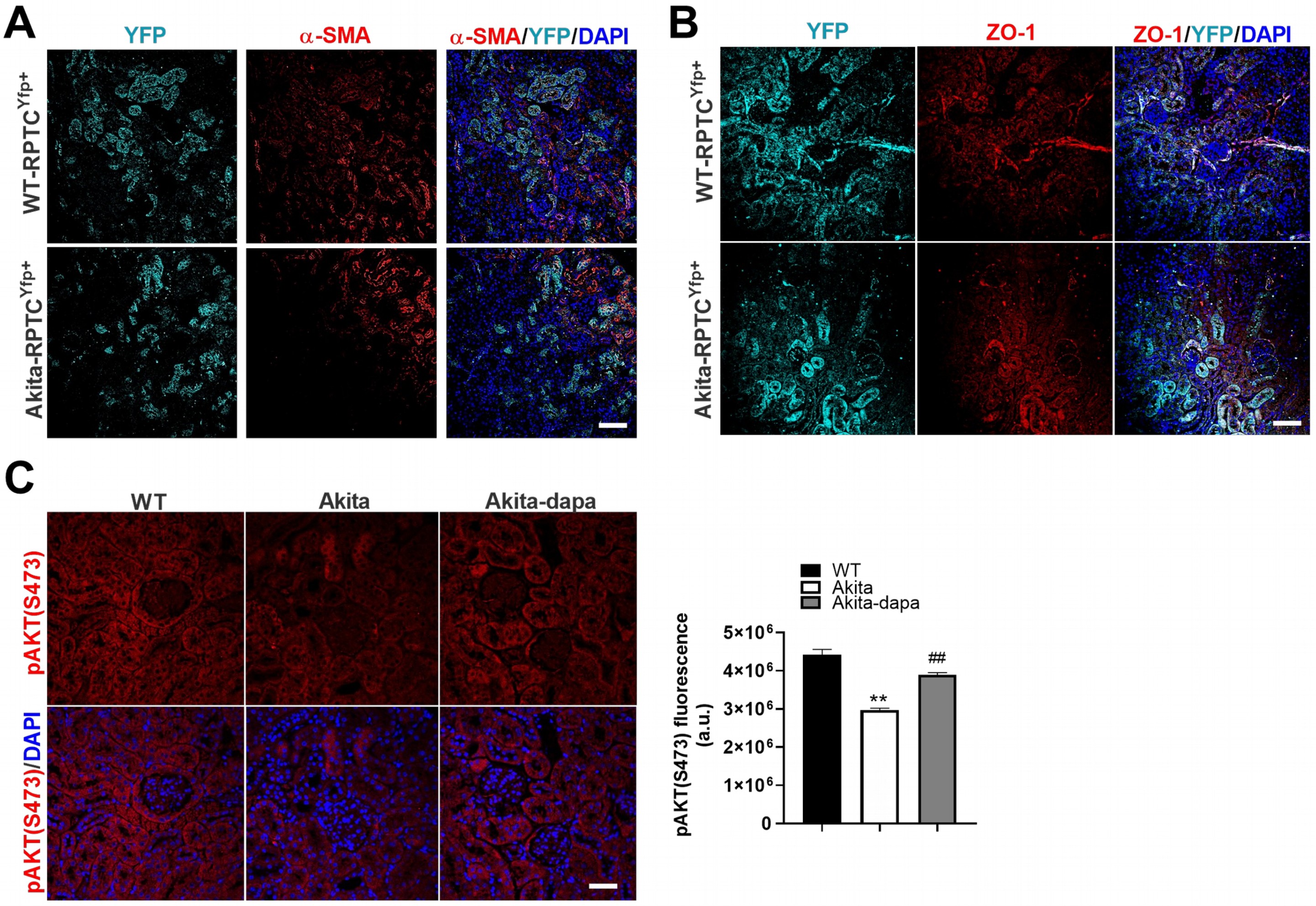
A
YFP
α-SMA
α-SMA/YFP/DAPI
WT-RPTCYfp+
Akita-RPTCYfp+
B
YFP
ZO-1
ZO-1/YFP/DAPI
WT-RPTCYfp+
Akita-RPTCYfp+
C
WT
Akita
Akita-dapa
pAKT(S473)
pAKT(S473)/DAPI
WT
Akita
Akita-dapa
pAKT(S473) fluorescence (a.u.)
5×10⁶
4×10⁶
3×10⁶
2×10⁶
1×10⁶
0
**
##

Figure S3. **Assessment of epithelial to mesenchymal transition (EMT) in DN. Related to Figure 2.** Analysis was performed on renal sections of 8-week old *Akita* and wildtype mice, as well as on renal sections of wildtype and *Akita Sglt2-cre;Rosa26-YFP+* reporter mice. (A-B) Immunofluorescence staining for YFP and the mesenchymal marker α-SMA (A), and the epithelial marker ZO-1 (B). (C) Immunofluorescence staining for pAKT (S473) in kidneys of WT and *Akita* mice treated with and without dapagliflozin. Scale bar, 50 μm. Data represent the mean ± SEM for 3 mice per group. $^{**}P<0.01$ relative to the wildtype control group; $^{\#\#}P<0.01$ relative to the untreated *Akita* group.

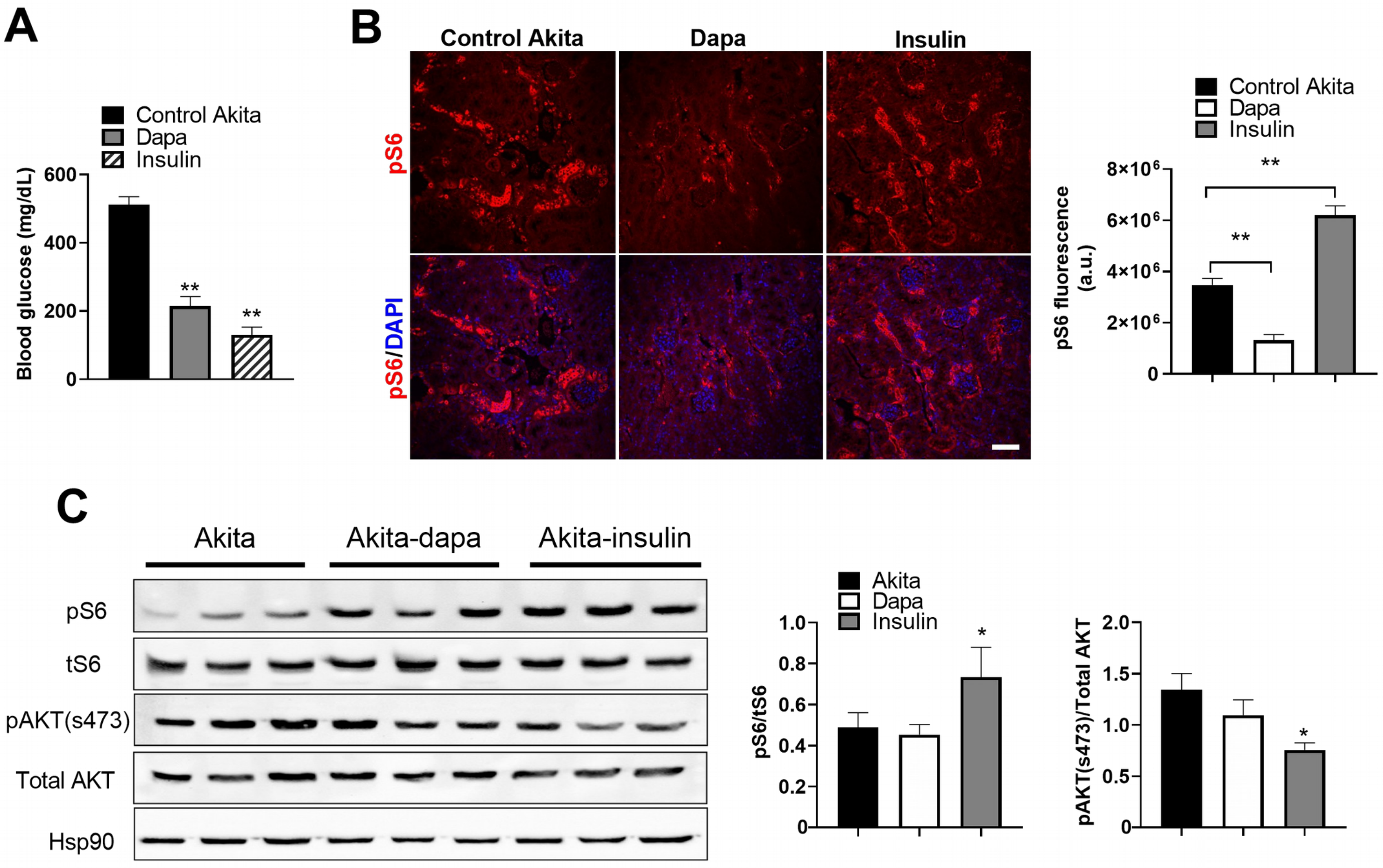

A
Control Akita
Dapa
Insulin
Blood glucose (mg/dL)
600
400
200
0
**
**
B
Control Akita
Dapa
Insulin
pS6
pS6/DAPI
Control Akita
Dapa
Insulin
pS6 fluorescence (a.u.)
8×10^6
6×10^6
4×10^6
2×10^6
0
**
**
C
Akita
Akita-dapa
Akita-insulin
pS6
tS6
pAKT(s473)
Total AKT
Hsp90
Akita
Dapa
Insulin
pS6/tS6
1.0
0.8
0.6
0.4
0.2
0
*
pAKT(s473)/Total AKT
2.0
1.5
1.0
0.5
0
*

Figure S4. **Effects of 5-day treatment with dapagliflozin or insulin on glycemia and mTORC1 activity in RPTCs. Related to Figure 2.** Diabetic *Akita* mice were treated with or without dapagliflozin (10 mg/kg/day in drinking water) or by SC injection of degludec insulin (4-6 U/day) for 5 days. (A) Blood glucose before and after treatment, (B) immunofluorescence for pS6 and quantifications of pS6 fluorescence intensity. (C) Western blotting for pS6 and phospho-AKT (s473) in whole kidney homogenates. Scale bar, 50 μm. Data represent the mean ± SEM of 3 mice per group. *P<0.05, **P<0.01 relative to the control untreated *Akita* group.

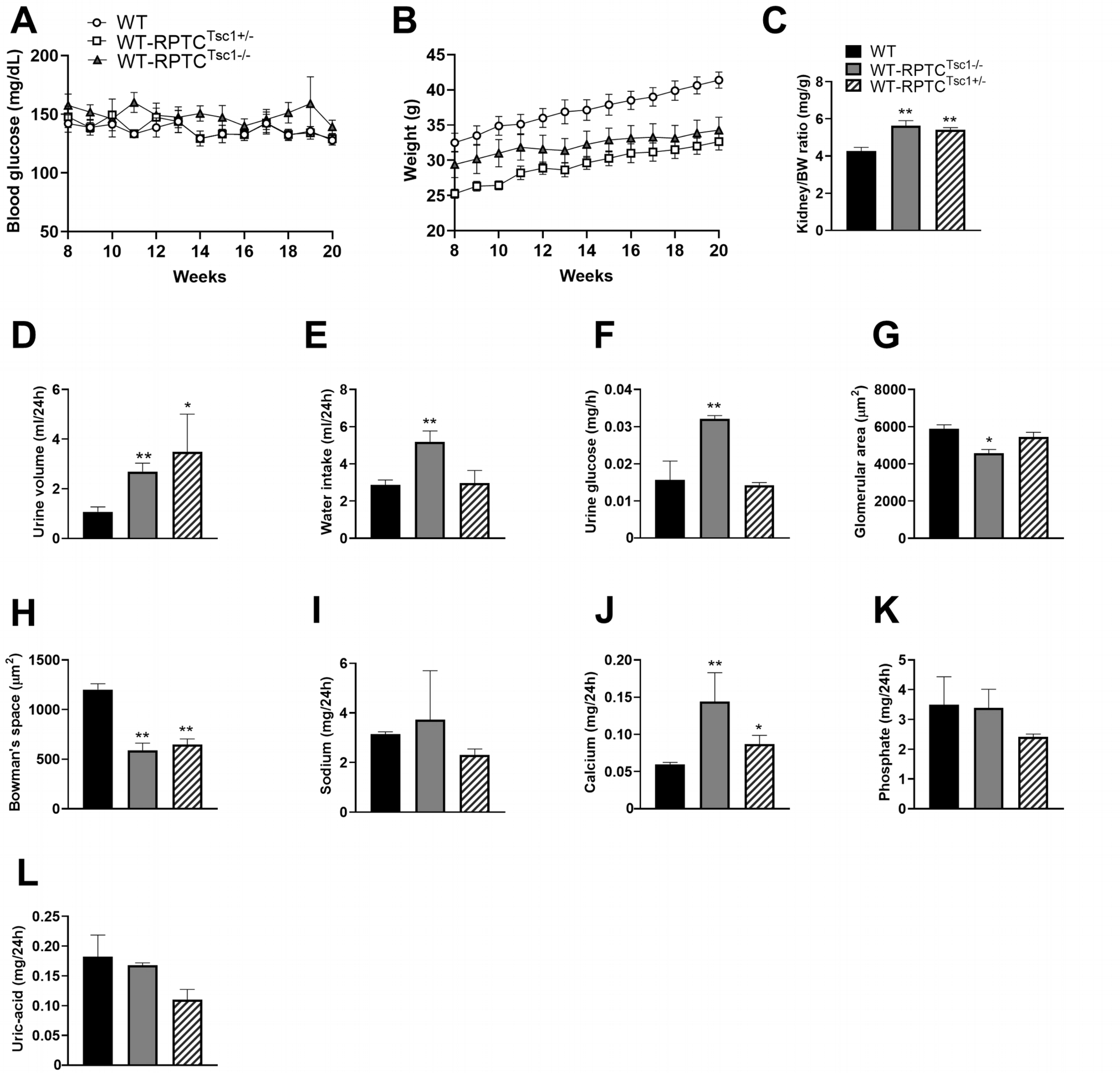
A
WT
WT-RPTC$^{Tsc1+/-}$
WT-RPTC$^{Tsc1-/-}$
Blood glucose (mg/dL)
Weeks
B
Weight (g)
Weeks
C
WT
WT-RPTC$^{Tsc1-/-}$
WT-RPTC$^{Tsc1+/-}$
Kidney/BW ratio (mg/g)
D
Urine volume (ml/24h)
E
Water intake (ml/24h)
F
Urine glucose (mg/h)
G
Glomerular area (μm^2)
H
Bowman's space (μm^2)
I
Sodium (mg/24h)
J
Calcium (mg/24h)
K
Phosphate (mg/24h)
L
Uric-acid (mg/24h)

Figure S5. **Metabolic effects of constitutive activation of mTORC1 by conditional *Tsc1* knockout in RPTCs. Related to Figure 4.** (A) Blood glucose, (B) body weight, (C) kidney to body weight ratio, (D-E) 24 h urine volume and water intake, (F) urine glucose concentration, (G-H) glomerular and Bowman's space cross-sectional areas, (I-L) urinary excretion of sodium, calcium, phosphate and uric acid. Data represent the mean ± SEM of 4 mice per group. *P<0.05, **P<0.01 relative to the wildtype control group.

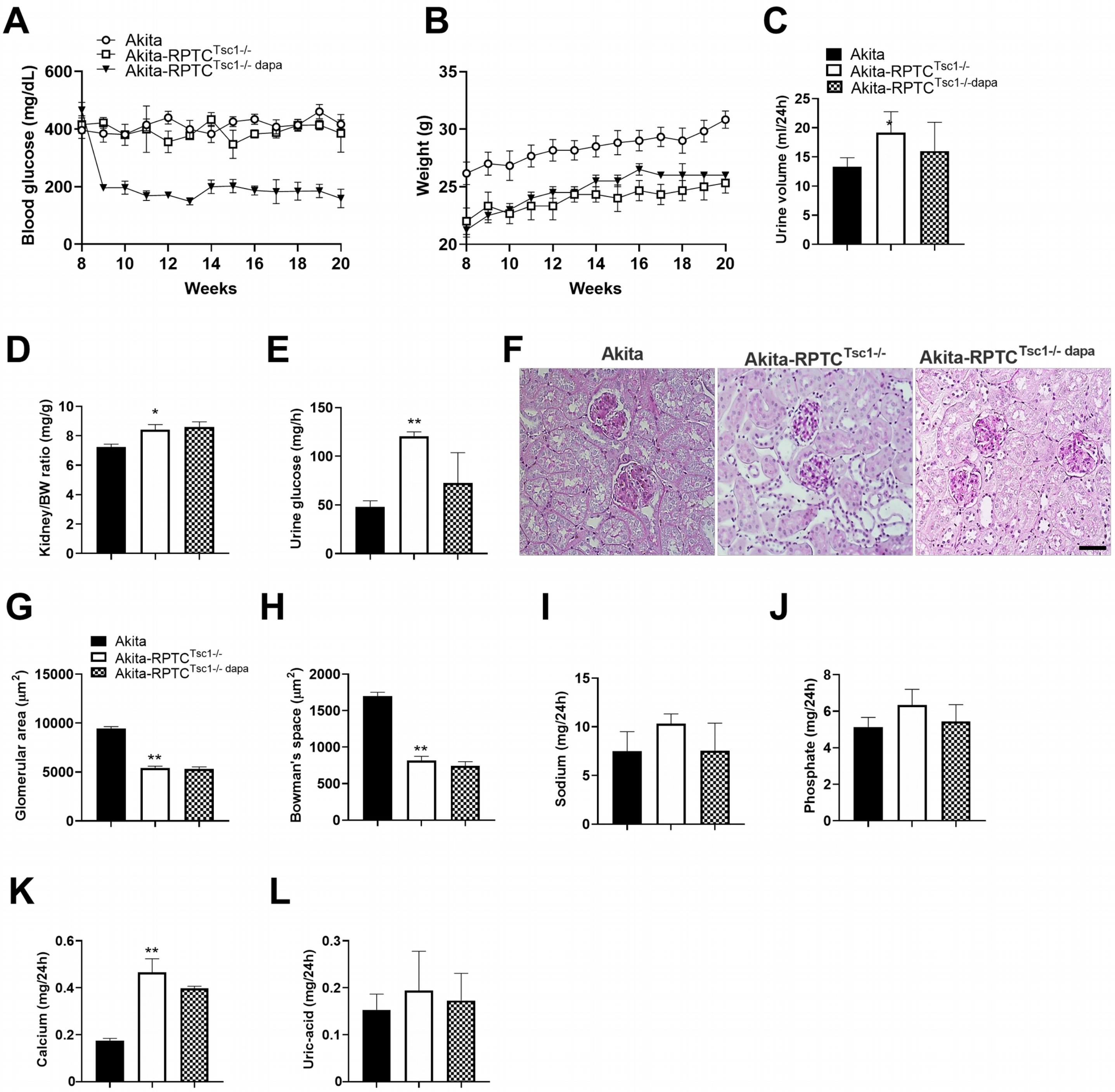
A
Akita
Akita-RPTC$^{Tsc1-/-}$
Akita-RPTC$^{Tsc1-/- dapa}$
Blood glucose (mg/dL)
Weeks
B
Weight (g)
Weeks
C
Urine volume (ml/24h)
D
Kidney/BW ratio (mg/g)
E
Urine glucose (mg/h)
F
G
Glomerular area (μm^2)
H
Bowman's space (μm^2)
I
Sodium (mg/24h)
J
Phosphate (mg/24h)
K
Calcium (mg/24h)
L
Uric-acid (mg/24h)

Figure S6. **Metabolic characterization of *RPTC-Tsc1fl/fl -Akita*-mice treated with or without dapagliflozin. Related to Figure 5.** (A) Blood glucose, (B) body weight, (C) 24 h urine volume, (D) kidney to body weight ratio, (E) urine glucose concentration. (F-H) A representative PAS staining and quantifications of glomerular and Bowman's space cross-sectional areas. (I-L) Urinary excretion of sodium, phosphate, calcium and uric acid. Scale bar, 50 µm. Data represent the mean ± SEM of 3-4 mice per group. *P<0.05, **P<0.01 relative to the control *Akita* group.

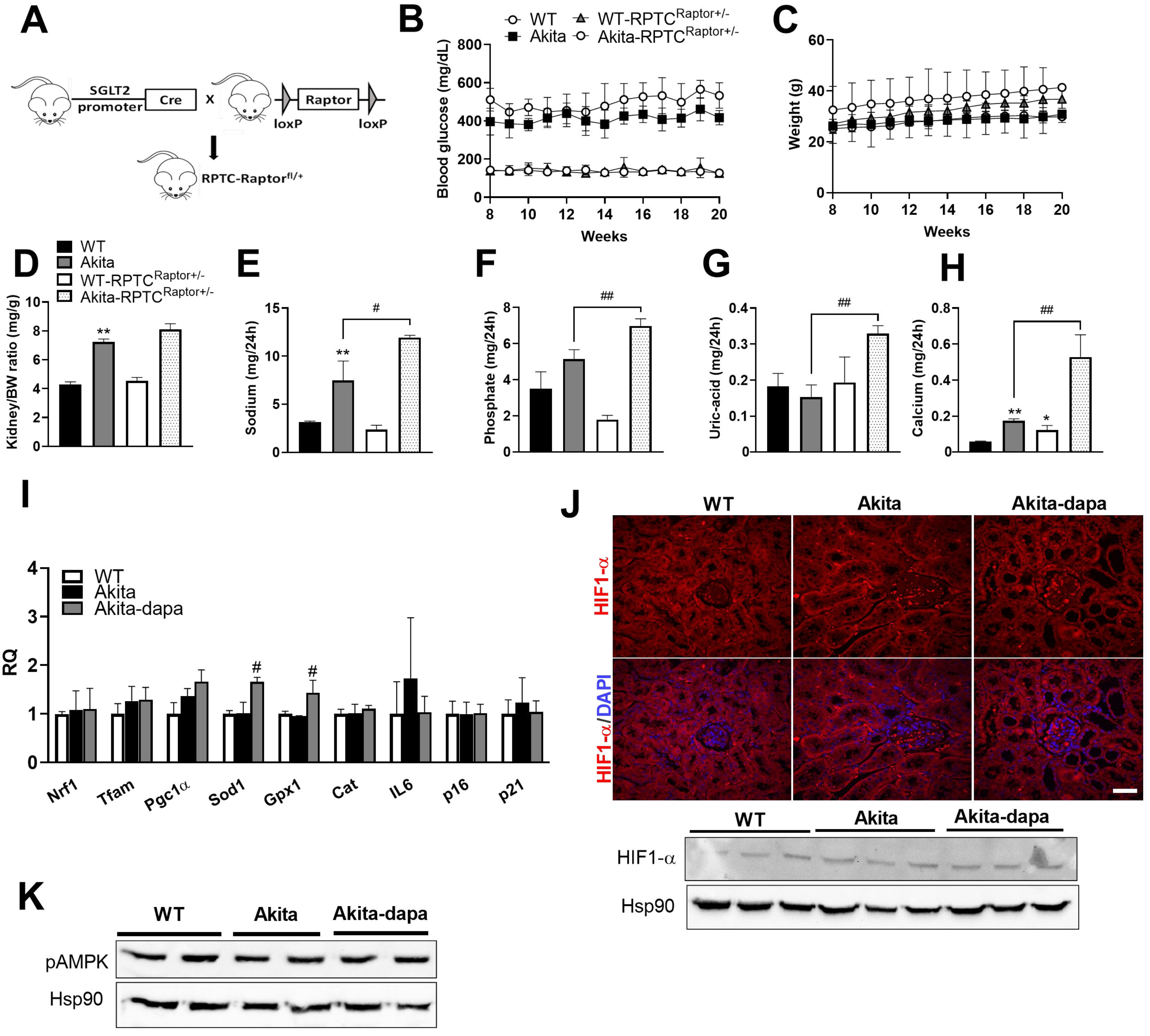

A
SGLT2 promoter
Cre
x
Raptor
loxP
loxP
RPTC-Raptorfl/+
B
WT
Akita
WT-RPTCRaptor+/-
Akita-RPTCRaptor+/-
Blood glucose (mg/dL)
Weeks
C
Weight (g)
Weeks
D
WT
Akita
WT-RPTCRaptor+/-
Akita-RPTCRaptor+/-
Kidney/BW ratio (mg/g)
E
Sodium (mg/24h)
F
Phosphate (mg/24h)
G
Uric-acid (mg/24h)
H
Calcium (mg/24h)
I
WT
Akita
Akita-dapa
RQ
Nrf1
Tfam
Pgc1α
Sod1
Gpx1
Cat
IL6
p16
p21
J
WT
Akita
Akita-dapa
HIF1-α
HIF1-α/DAPI
HIF1-α
Hsp90
K
WT
Akita
Akita-dapa
pAMPK
Hsp90

Figure S7. **Metabolic effects of conditional *Raptor* knockout in RPTCs (A-H)**. **Effects of diabetes and of treatment with dapagliflozin on the expression and activity of genes regulating mitochondrial biogenesis, oxidative stress, hypoxia and senescence (I-K). Related to Figure 6.** (A-H) Analyses were performed in *RPTC-Raptorfl/+* compared to control *Akita* and wildtype mice. (A) Schematic diagram of the Cre-mediated recombination strategy for generation of heterozygous Raptor knockout in RPTCs. (B) Blood glucose, (C) body weight, (D) kidney to body weight ratio, (E-H) urinary excretion of sodium, phosphate, uric acid and calcium. (I) Gene expression analysis by qPCR. (J) immunostaining and Western blotting for HIF1α. (K) Western blotting for pAMPK. Data represent the mean ± SEM of 4-6 mice per group. *P<0.05, **P<0.01 relative to the wildtype control group. $^{\#}$P<0.05, $^{\#\#}$P<0.01 relative to the control *Akita* group.
